# The Neglected Role of GSDMD C‐Terminal in Counteracting Type I Interferon Signaling

**DOI:** 10.1002/advs.202505255

**Published:** 2025-06-20

**Authors:** Weilv Xu, Suhui He, Wen Shi, Jinxia Xu, Shiyang Liu, Zexu Yu, Danyue Li, Qiao Jin, Yumeng Wang, Zian Zhang, Qian Lv, Yuanxiang Ge, Yunjie Li, Xinyue Li, Nan Chen, Xinyu Fu, Yang Yang, Fushan Shi

**Affiliations:** ^1^ MOA Key Laboratory of Animal Virology Zhejiang University Center for Veterinary Sciences Zhejiang University Hangzhou 310058 China; ^2^ Department of Veterinary Medicine College of Animal Sciences Zhejiang University Hangzhou 310058 China; ^3^ College of Animal Science and Technology Guangxi University Nanning 530004 China; ^4^ Key Laboratory of Applied Technology on Green‐Eco‐Healthy Animal Husbandry of Zhejiang Province College of Animal Science and Technology & College of Veterinary Medicine Zhejiang A&F University Hangzhou 311300 China; ^5^ Zhejiang University‐Xinchang Joint Innovation Centre (TianMu Laboratory) Zhejiang University Shaoxing 312500 China

**Keywords:** autophagic degradation, GSDMD‐CT, innate immunity, RIG‐I, TBK1, TRIM28

## Abstract

The GSDMD N‐terminal fragment (GSDMD‐NT)‐mediated pyroptosis is extensively investigated. However, the role of the C‐terminal domain of GSDMD (GSDMD‐CT) is unexplored. This study demonstrates that GSDMD‐CT acts as a negative regulator that suppresses IFN‐I signaling during viral infection. Mechanistically, GSDMD‐CT, released upon virus infection, interacts separately with retinoic acid‐inducible gene I (RIG‐I) and tank‐binding kinase (TBK1), promoting the selective autophagic degradation of RIG‐I via K48‐linked polyubiquitination at Lys181 and TBK1 via K27‐linked polyubiquitination at Lys487 by the E3 ligase TRIM28, which serves as a recognition signal for the cargo receptor NDP52 and TOLLIP, respectively. Moreover, the P414, Q416, and E459 amino sites are crucial for GSDMD‐CT in counteracting antiviral responses. The findings highlight the role of GSDMD‐CT in inhibiting antiviral immunity, providing insights into how viruses manipulate host defense mechanisms to enhance infection.

## Graphical abstract

The C‐terminal domain of Gasdermin D (GSDMD‐CT) shows functions beyond pyroptosis. GSDMD‐CT negatively regulates IFN‐I signaling during viral infection by triggering autophagic degradation of RIG‐I and TBK1. GSDMD‐CT interacts with retinoic acid‐inducible gene‐I (RIG‐I) and tank‐binding kinase 1 (TBK1), leading to TRIM28‐mediated ubiquitination and autophagy. This study identifies GSDMD‐CT as an important suppressor of antiviral immunity, revealing how viruses can manipulate host autophagy pathways.

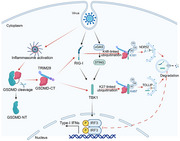

## 1 Introduction

Pyroptosis, a programmed form of necrotic cell death, is characterized by cellular swelling and the expulsion of large bubbles from the plasma membrane, garnering increasing attention due to its association with innate immunity.^[^ ^1^, ^2^, ^3^ ^]^ Governed by the assembly and activation of inflammasomes, pyroptosis involves canonical inflammasomes such as NLR Family Pyrin Domain Containing 1 (NLRP1), NLR Family Pyrin Domain Containing 3 (NLRP3), NLR family caspase activation and recruitment domain‐containing 4 (NLRC4), and Absent In Melanoma 2 (AIM2), which assemble in the cytosol to recruit and activate Caspase‐1. Conversely, noncanonical inflammasomes are triggered by human Caspase‐4/5 (or mouse orthologs Caspase‐11), which directly bind to intracellular lipopolysaccharide (LPS).^[^ ^4^, ^5^, ^6^ ^]^ Gasdermins, serving as the executors of pyroptosis, have garnered significant research attention, particularly Gasdermin D (GSDMD).^[^ ^2^ ^]^ GSDMD can be cleaved by Caspase‐1/4/5/11, resulting in the release of its N‐terminal domain (GSDMD‐NT) and C‐terminal domain (GSDMD‐CT). While existing research has primarily focused on the function of GSDMD‐NT, which can oligomerize and form pores in the cell membrane, leading to the release of inflammatory cytokines and the initiation of pyroptosis, the biological function of GSDMD‐CT remains unexplored. It is only known that GSDMD‐CT may interact with GSDMD‐NT, leading to the autoinhibition of GSDMD, which is released upon interdomain cleavage by inflammatory Caspases.^[^ ^4^, ^7^, ^8^, ^9^ ^]^ Although it has been shown that overexpression of GSDMD‐CT can block LPS‐induced pyroptosis in HeLa cells by trans‐inhibiting the endogenous GSDMD‐NT generated from Caspase‐4 cleavage, other studies have found that GSDMD‐CT overexpression has no effect on pyroptosis.^[^ ^10^ ^]^

Numerous pathogens have been identified to trigger the activation of inflammasomes. Severe Acute Respiratory Syndrome Coronavirus 2 (SARS‐CoV‐2) protease 3CL cleaves NLRP1, leading to NLRP1 inflammasome assembly and activation.^[^ ^11^ ^]^ Influenza A virus (IAV) infection activates the AIM2 inflammasome, contributing significantly to lung injury and mortality induced by IAV.^[^ ^12^ ^]^ The N protein of SARS‐CoV‐2 interacts with the NLRP3 protein, promoting the binding of NLRP3 with ASC and facilitating NLRP3 inflammasome assembly.^[^ ^13^ ^]^ Influenza virus M2 protein stimulates NLRP3 inflammasome activation.^[^ ^14^ ^]^ Additionally, the EMCV (Encephalomyocarditis Virus) 2B protein induces NLRP3 inflammasome activation by triggering Ca^2+^ flux from intracellular stores to the cytosol.^[^ ^15^ ^]^ Moreover, the EV71 (Enterovirus 71) 3D protein promotes NLRP3 inflammasome activation at the early stage of infection, whereas viral proteases 2A and 3C counteract inflammasome activation by cleaving NLRP3 at a later stage of infection.^[^ ^16^, ^17^ ^]^ In *Enterohemorrhagic Escherichia coli* (EHEC) infection, Caspase‐11 is activated via a noncanonical inflammasome pathway, leading to the cleavage of GSDMD and the generation of an active pore‐forming N‐terminal fragment (GSDMD‐NT). However, Caspase‐3, activated by EHEC Stx, subsequently cleaves the GSDMD‐NT produced by Caspase‐11, rendering it nonfunctional.^[^ ^18^ ^]^ While it is undeniable that inflammasome activation plays a critical role in defending against viral infections, the possibility of viruses utilizing sophisticated regulatory mechanisms to counteract the antiviral effects of inflammasomes remains unexplored.

Given the prevalent involvement of GSDMD as a downstream effector across multiple inflammasomes, its role in modulating the type I interferon response warrants scrutiny. However, existing research presents conflicting findings. While initial studies suggest that GSDMD suppresses the cGAS‐driven type I interferon response to cytosolic DNA and *Francisella novicida* in macrophages by inducing intracellular potassium (K^+^) depletion through membrane pores,^[^ ^19^ ^]^ subsequent investigations indicate its potential to facilitate the noncanonical secretion of beta interferon (IFN‐β), thereby augmenting the responses of interferon‐stimulated genes (ISGs).^[^ ^20^ ^]^ The new study also highlights the pivotal role of GSDMD in promoting lung neutrophil responses, thus amplifying inflammation and pathogenesis induced by the influenza virus.^[^ ^21^ ^]^

Here, our study demonstrates that full‐length GSDMD (GSDMD‐FL) promotes the type I interferon response, while the C‐terminal fragment of GSDMD (GSDMD‐CT) inhibits it. Mechanically, GSDMD‐CT interacts with retinoic acid‐inducible gene I (RIG‐I) and tank‐binding kinase (TBK1), thereby promoting K48‐linked polyubiquitination at Lys181 of RIG‐I and K27‐linked polyubiquitination at Lys487 of TBK1 via E3 ligase TRIM28, which acts as a recognition signal for the cargo receptor NDP52 and TOLLIP and leads the selective autophagic degradation of both RIG‐I and TBK1. P414, Q416, and E459 amino acid sites are crucial for GSDMD‐CT in attenuating the type I interferon response. Our study identifies GSDMD‐CT as a negative regulator in antiviral innate immunity and offers a perspective on the potential for viruses to activate pyroptosis to evade host antiviral response, thereby shedding light on virus infection control and disease therapy.

## 2 Results

### 2.1 GSDMD‐KO Mice Exhibit Stronger Resistance to EMCV Infection

**Figure 1:**
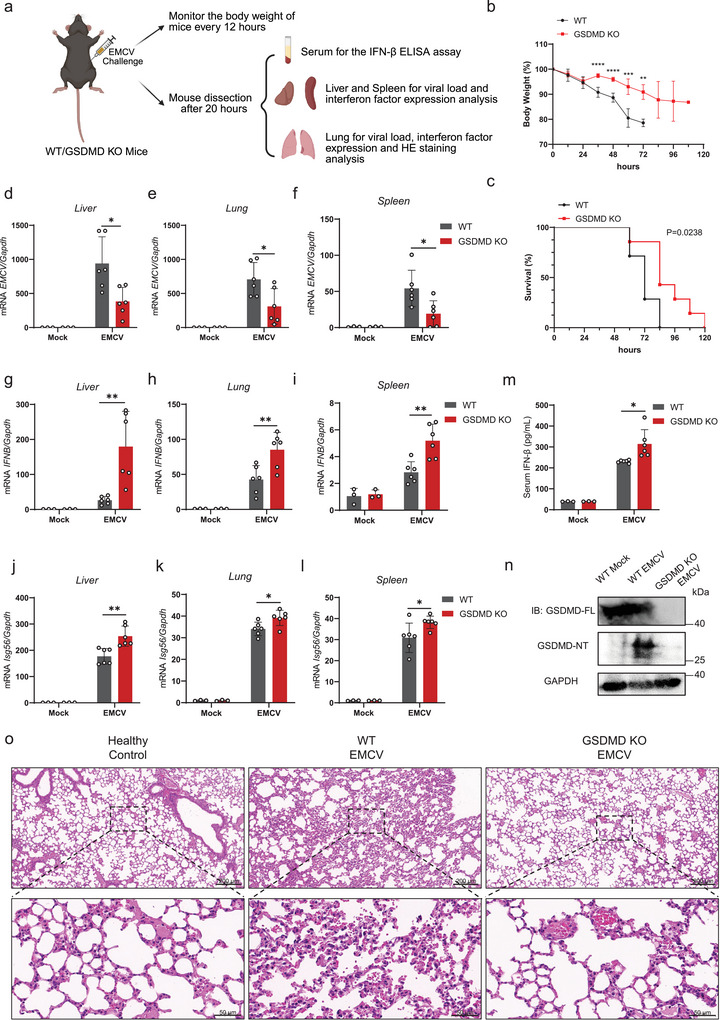
GSDMD‐KO mice exhibit stronger resistance to EMCV infection. a) Schematic of the approach and strategy used to investigate the effect of GSDMD deficiency in EMCV infection. b) Weight of WT and GSDMD‐KO mice (*n* = 7 mice per group) after intraperitoneal injection of EMCV (1 × 10^4^ PFU per mouse). ^****^ *p* < 0.0001, ^***^ *p* < 0.001, ^**^ *p* < 0.01 (Student's *t*‐test). c) Survival of WT and GSDMD‐KO mice (*n* = 7 mice per group) after intraperitoneal injection of EMCV (1 × 10^4^ PFU per mouse). Log‐rank (Mantel‐Cox) test. d–l) RT‐PCR analysis of EMCV (d–f), *IFNB* (g–i), *Isg56* (j–l) mRNA levels in the liver (left), lungs (center), and spleen (right) from WT or GSDMD‐KO mice treated with phosphate‐buffered saline (PBS) or infected with EMCV (1 × 10^6^ PFU per mouse) via intraperitoneal injection for 20 h. ^**^ *p* < 0.01, ^*^ *p* < 0.05 (Student's *t*‐test). m) ELISA for IFN‐β in the serum of mice, as in (d–l). ^*^ *p* < 0.05 (Student's *t*‐test). n) GSDMD protein levels of the lung tissues were detected by immunoblot analysis. o) Representative hematoxylin and eosin (H&E)‐stained images of lung sections from mice as described in (d–l). Scale bars, 200 µm (top) and 50 µm (bottom).

To investigate the roles of GSDMD during virus infection in vivo, we infected wild‐type (WT) and GSDMD‐knock‐out (GSDMD‐KO) mice with EMCV by intraperitoneal injection (**Figure** **1**a). The genotyping results of GSDMD‐KO mice are shown in Figure S1a,b (Supporting Information). While the GSDMD‐KO mice infected with 1 × 10^6^ PFU EMCV did not exhibit significant weight loss compared to WT mice (Figure S1c, Supporting Information), they demonstrated improved survival (Figure S1d, Supporting Information). Additionally, GSDMD deficiency improved both weight loss and survival in mice infected with 1 × 10^4^ PFU EMCV (Figure 1b,c). We then examined the replication of EMCV in the liver, lung, and spleen and found that viral proliferation in organs from GSDMD‐KO mice was significantly reduced compared to WT mice (Figure 1d–f). Consistently, the transcriptional levels of *IFNB* and *Isg56* in GSDMD‐KO mice, as well as the production of IFN‐β in serum, were significantly higher than those in the WT group (Figure 1g–m). Immunoblotting analysis of lung tissue lysates from WT mice infected with EMCV demonstrated the cleavage of GSDMD (Figure 1n). This finding suggests that EMCV infection triggers GSDMD processing and pyroptosis in the lungs. Histopathological examination of lung tissues from GSDMD‐KO mice infected with EMCV revealed a significant reduction in pulmonary inflammation compared to infected WT mice. This observation supports the role of GSDMD in contributing to the inflammatory response observed during EMCV infection (Figure 1o). Taken together, these data demonstrate that GSDMD deficiency protects mice from EMCV infection and promotes IFN‐I signaling in vivo.

### 2.2 GSDMD‐FL Promotes IFN‐I Immune Response upon Viral Infection

**Figure 2:**
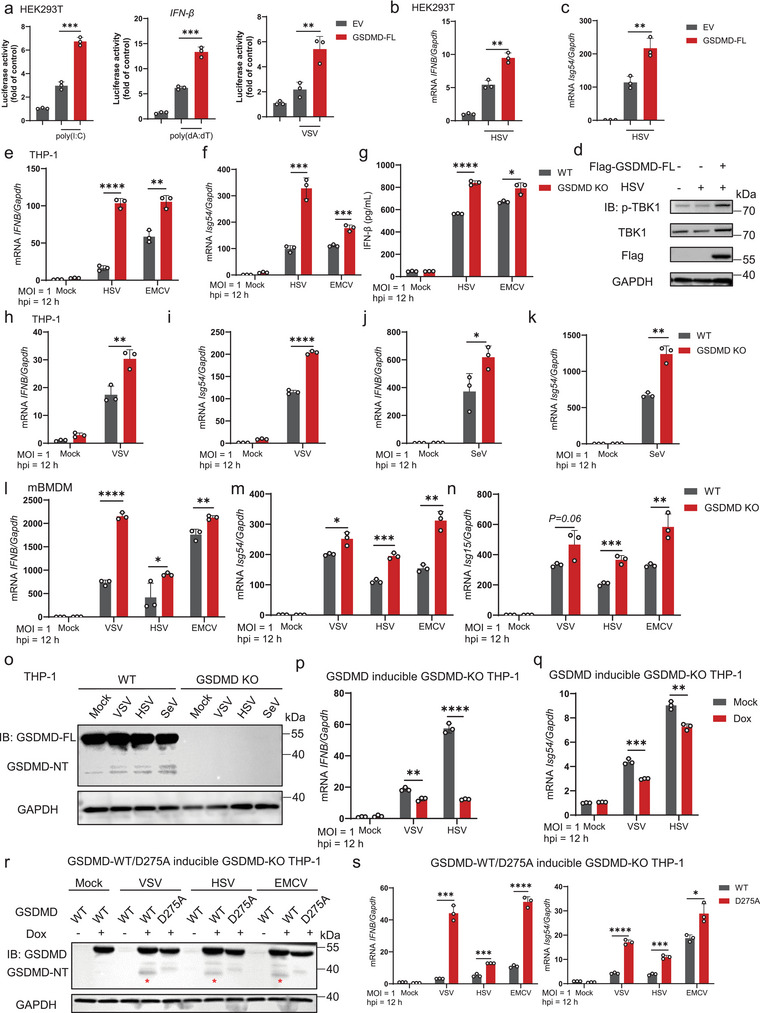
GSDMD‐FL promotes IFN‐I immune response upon viral infection. a) Luciferase reporter assays analyzing *IFN‐β* promoter activity of HEK293T cells transfected with GSDMD‐full length (GSDMD‐FL) or empty vector (EV) for 24 h, followed by treatment with or without poly(I:C), poly(dA:dT) or VSV (MOI = 1) for 12 h, respectively. Data are represented as mean ± SD. ^***^ *p* < 0.001, ^**^ *p* < 0.01 (Student's *t*‐test). b,c) RT‐PCR analysis of *IFNB* (b) and *Isg54* (c) mRNA levels in HEK293T cells transfected with GSDMD‐FL or EV for 24 h, followed by HSV (MOI = 0.1) infection for 12 h. Data are represented as mean ± SD. ^***^ *p* < 0.001, ^*^ *p* < 0.05 (Student's *t*‐test). d) HEK293T cells were transfected with Flag‐GSDMD‐FL or EV for 24 h, followed by HSV (MOI = 0.1) infection for 12 h. Cell lysates were collected for immunoblot analysis with indicated antibodies. e–k) PMA‐differentiated WT or GSDMD‐knockout (GSDMD‐KO) THP‐1 cells were treated with HSV (MOI = 1) or EMCV (MOI = 1) or VSV (MOI = 1) or SeV (MOI = 1) for 12 h. *IFNB* or *Isg54* mRNA level was detected by RT‐PCR analysis (e, f, and h–k), and IFN‐β release in the supernatants was determined by ELISA analysis (g). Data are represented as mean ± SD. ^****^ *p* < 0.0001, ^***^ *p* < 0.001, ^**^ *p* < 0.01, ^*^ *p* < 0.05 (Student's *t*‐test). l–n) WT or GSDMD‐knockout (GSDMD‐KO) BMDM cells were treated with VSV (MOI = 1) or HSV (MOI = 1) or EMCV (MOI = 1) for 12 h. *IFNB* or *Isg54* or *Isg15* mRNA levels were detected by RT‐PCR analysis. Data are represented as mean ± SD. ^****^ *p* < 0.0001, ^***^ *p* < 0.001, ^**^ *p* < 0.01, ^*^ *p* < 0.05 (Student's *t*‐test). o) Cell lysates from E‐K were collected for immunoblot analysis. p,q) RT‐PCR analysis of *IFNB* and *Isg54* mRNA levels in GSDMD‐WT‐inducible GSDMD‐KO THP‐1 cells treated with Dox (500 ng mL^−1^) for 72 h, followed by PMA (1 µm) treatment for 24 h and then VSV (MOI = 1) or HSV (MOI = 1) infection for 12 h. Data are represented as mean ± SD. ^****^ *p* < 0.0001, ^***^ *p* < 0.001, ^**^ *p* < 0.01 (Student's *t*‐test). r, s) RT‐PCR analysis of *IFNB* and *Isg54* mRNA levels in GSDMD‐WT‐inducible GSDMD‐KO THP‐1 cells or GSDMD‐D275A‐inducible GSDMD‐KO THP‐1 cells treated with Dox (500 ng mL^−1^) for 72 h, followed by PMA (1 µm) treatment for 24 h and then VSV (MOI = 1) or HSV (MOI = 1) or EMCV (MOI = 1) infection for 12 h. Data are represented as mean ± SD. ^****^ *p* < 0.0001, ^***^ *p* < 0.001, ^**^ *p* < 0.01, ^*^ *p* < 0.05 (Student's *t*‐test). Cell lysates were collected for immunoblot analysis with indicated antibodies (r). Red asterisks marked the position of the cleaved GSDMD bands.

We then endeavored to elucidate the role of GSDMD‐FL in type I interferon immunity. Through the overexpression of GSDMD‐FL in HEK293T cells and subsequent performance of the *IFN‐β* luciferase reporter assay, we discovered that GSDMD‐FL significantly enhanced the activation of *IFN‐β* induced by intracellular poly(I:C), poly(dA:dT), or vesicular stomatitis virus (VSV) (**Figure** **2**a). Consistently, ectopic expression of GSDMD‐FL markedly increased the transcription of antiviral genes, including *IFNB* and *Isg54*, in HEK293T cells infected with Herpes simplex virus (HSV) (Figure 2b,c). Furthermore, GSDMD‐FL overexpression led to enhanced phosphorylation of TBK1 (Figure 2d). Similar results were observed in HEK293T cells stimulated with poly(I:C) or poly(dA:dT), as well as in cells infected with VSV or Sendai virus (SeV) (Figure S2a–f, Supporting Information).

However, upon infecting GSDMD‐KO THP‐1 cells with VSV, HSV, EMCV, and SeV, we observed the GSDMD deficiency remarkably promoted the transcription of *IFNB* and *Isg54*, as well as the secretion of IFN‐β (Figure 2e–k). To confirm these results, bone marrow‐derived macrophages (BMDMs) were isolated from both WT and GSDMD‐KO mice, yielding similar findings (Figure 2l–n). This was in stark contrast to the results obtained from HEK293T cells but was consistent with the results we found in mice infected with EMCV (Figure 1). Given that we observed GSDMD cleavage during EMCV infection (Figure 1n), a finding also evident in IAV infection,^[^ ^21^ ^]^ and considering that HEK293T cells, which lack inflammasome components, do not undergo GSDMD cleavage upon viral infection (Figure S2g, Supporting Information), we hypothesized that the enhanced IFN‐I signaling observed in GSDMD‐KO mice might be attributed to GSDMD cleavage fragments. Consistently, we also observed cleavage of GSDMD upon infection with these viruses (Figure 2o), which suggested that the protective function observed in GSDMD deficiency was due to the absence of GSDMD cleavage fragments. We also found that cleavage levels of GSDMD increased in response to both higher viral titers and longer infection durations (Figure S2h, Supporting Information).

To clarify that the enhancement effects of GSDMD deficiency are direct and not due to an indirect effect of cell lysis, we used the osmoprotectant glycine to prevent pyroptosis‐related membrane rupture, thereby creating a “hyperactivation” scenario. Under these conditions, we found that GSDMD deficiency still notably promoted the transcription of *IFNB* and *Isg54* (Figure S2i,j, Supporting Information), suggesting that this phenomenon is independent of cell lysis. Consequently, these findings suggest that GSDMD‐NT, which executes pyroptosis, may not be the key mediator of these transcriptional changes, pointing instead to a potential regulatory role for the GSDMD‐CT, which we explore further in this study.

Next, we generated doxycycline (Dox)‐inducible GSDMD‐FL‐WT cell line in GSDMD‐KO THP‐1 cells. To minimize the potential influence of Dox treatment, we first treated vector‐inducible GSDMD‐KO THP‐1 cells with or without Dox for 72 h, followed by VSV or HSV infection. The results showed that Dox treatment had no effect on the transcription of *IFNB*, *Isg54*, and *Isg56* (Figure S2k–m, Supporting Information). Upon reconstitution with GSDMD‐FL‐WT, the cells exhibited decreased mRNA levels of *IFNB* and *Isg54* upon viral infection (Figure 2p,q), correlating with the presence of cleavage fragments. Considering the D275 residue serves as the cleavage site, we generated a GSDMD‐FL‐D275A Dox‐inducible cell line in GSDMD‐KO THP‐1 cells. Initially, we primed the cells with LPS for 4 h and subsequently stimulated them with nigericin for 1 h to validate the significance of the D275 residue site for pyroptosis (Figure S2o,p, Supporting Information). Following the Dox treatment, we infected the cells with VSV, HSV, or EMCV. The results showed that cells expressing GSDMD‐FL‐D275A, a mutant that cannot be cleaved into two fragments (Figure 2r), exhibited increased transcription levels of antiviral genes and a higher secretion of IFN‐β (Figure 2s; Figure S2q, Supporting Information). Since GSDMD‐NT is known to promote the unconventional release of IFN‐β,^[^ ^20^ ^]^ we hypothesized that the other fragment, GSDMD‐CT, is responsible for negatively regulating IFN‐I signaling.

### 2.3 GSDMD‐CT Attenuates IFN‐I Immune Signaling Activation

**Figure 3:**
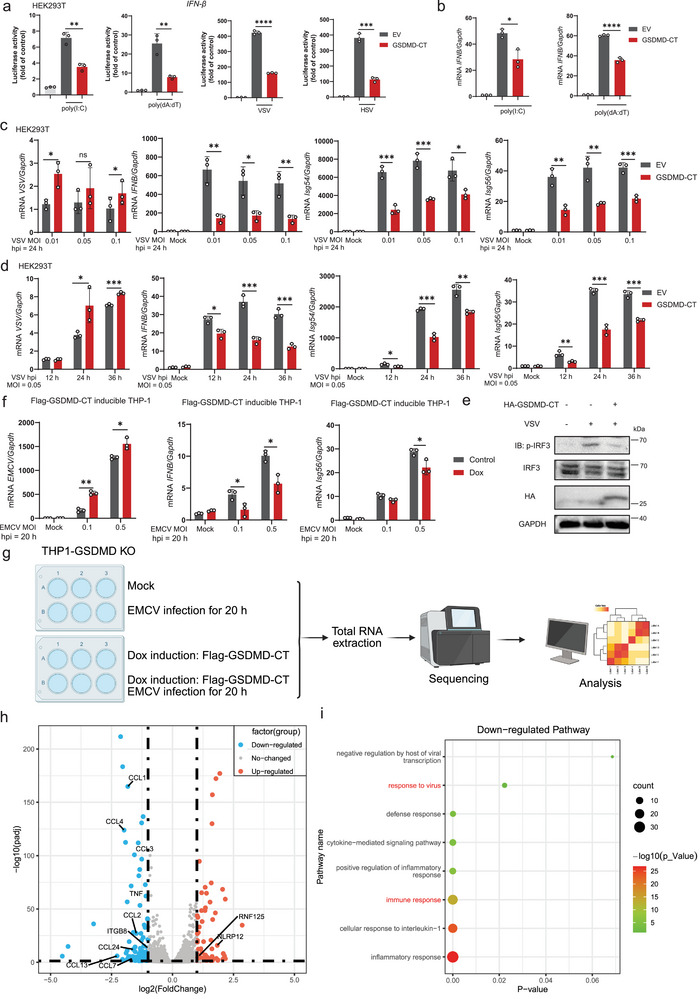
GSDMD‐CT attenuates IFN‐I immune signaling activation. a) Luciferase reporter assays analyzing *IFN‐β* promoter activity of HEK293T cells transfected with GSDMD‐CT or empty vector (EV) for 24 h, followed by treatment with or without poly(I:C), poly(dA:dT), VSV (MOI = 1) or HSV (MOI = 1) for 12 h. Data are represented as mean ± SD. ^****^ *p* < 0.0001, ^*^ *p* < 0.05 (Student's *t*‐test). b) RT‐PCR analysis of *IFNB* mRNA level in the HEK293T cells transfected with GSDMD‐CT or EV for 24 h, followed by treatment with or without poly(I:C) or poly(dA:dT) for 12 h. Data are represented as mean ± SD. ^****^ *p* < 0.0001, ^***^ *p* < 0.001, ^**^ *p* < 0.01 (Student's *t* test). c) RT‐PCR analysis of indicated mRNA levels in the HEK293T cells transfected with GSDMD‐CT or EV for 24 h, followed by VSV infection at the indicated MOI for 24 h. Data are represented as mean ± SD. ^***^ *p* < 0.001, ^**^ *p* < 0.01, ^*^ *p* < 0.05 (Student's *t*‐test). d) RT‐PCR analysis of indicated mRNA levels in the HEK293T cells transfected with GSDMD‐CT or EV for 24 h, followed by VSV infection (MOI = 0.05) for the indicated time. Data are represented as mean ± SD. ^***^ *p* < 0.001, ^**^ *p* < 0.01, ^*^ *p* < 0.05 (Student's *t*‐test). e) Immunoblot analysis of HEK293T cells transfected with HA‐GSDMD‐CT or EV for 24 h, followed by VSV infection (MOI = 0.05) for 24 h. f) RT‐PCR analysis of indicated mRNA levels in Flag‐GSDMD‐CT inducible THP‐1 cells treated with Dox (500 ng mL^−1^) for 72 h, followed by PMA (1 µm) treatment for 24 h and then EMCV (MOI = 1) infection for 12 h. Data are represented as mean ± SD. ^**^ *p* < 0.01, ^*^ *p* < 0.05 (Student's *t*‐test). g) Schematic of the approach and strategy used to identify differentially expressed genes in GSDMD‐CT inducible cells upon EMCV infection. h) Volcano plot of genes with differential expression after stimulation. Each dot represents an individual gene. Red dots denote upregulated DEGs, while blue dots represent downregulated DEGs. i) Gene ontology (GO) enrichment analysis of the DEGs.

Subsequently, we investigated the direct function of GSDMD‐CT. Our findings revealed that overexpression of GSDMD‐CT suppressed the activity of the *IFN‐β* promoter induced by poly(I:C), poly(dA:dT), VSV, or HSV in HEK293T cells (**Figure** **3**a). Additionally, we transfected GSDMD‐CT in HEK293T cells for 24 h, and stimulated them with poly(I:C), poly(dA:dT) for 12 h. The results demonstrated that ectopic expression of GSDMD‐CT reduced the mRNA levels of *IFNB*, *Isg54*, and *Isg56* (Figure 3b; Figure S3a,b, Supporting Information). Consistently, HEK293T cells transfected with GSDMD‐CT and subsequently infected with VSV at various multiplicities of infection (MOI) and hours post‐infection (hpi) showed enhanced viral replication and decreased *IFNB* transcription (Figure 3c,d). The phosphorylation of IRF3 was also reduced by the expression of GSDMD‐CT (Figure 3e). Experiments involving HSV and SeV infections yielded similar outcomes (Figure S3c,d, Supporting Information). Moreover, we generated Dox‐inducible GSDMD‐CT cell lines in both WT and GSDMD‐KO THP‐1 cells (Figure S3f, Supporting Information). Upon infecting THP‐1 cells induced with GSDMD‐CT with EMCV and VSV, we observed enhanced viral replication (Figure 3f) and attenuated IFN‐I signaling (Figure 3f; Figure S3e, Supporting Information) compared to cells without Dox induction. We also employed RNA‐seq to comprehensively investigate the differences between cells induced with GSDMD‐CT and those not induced when infected with EMCV (Figure 3g). Compared with control groups, GSDMD‐CT induction resulted in 184 differentially expressed genes (DEGs), including 70 upregulated and 114 downregulated genes, which were identified using the cutoff (|Log2 fold change| > 1 and adjusted *p*‐value < 0.05) (Figure S3g; Table S1, Supporting Information). We scrutinized the expression of several representative genes associated with the response to virus and immune response (Figure 3h; Figure S3h, Supporting Information), indicating that the expression of GSDMD‐CT dampened the immune response upon virus infection. Subsequent gene ontology (GO) analysis unveiled the extensive downregulation of genes associated with immune pathways, including response to virus and immune response (Figure 3i). In conclusion, our findings suggest that GSDMD‐CT attenuates the activation of IFN‐I immune signaling during virus infection.

### 2.4 GSDMD‐CT Promotes the Autophagic Degradation of RIG‐I and TBK1

**Figure 4:**
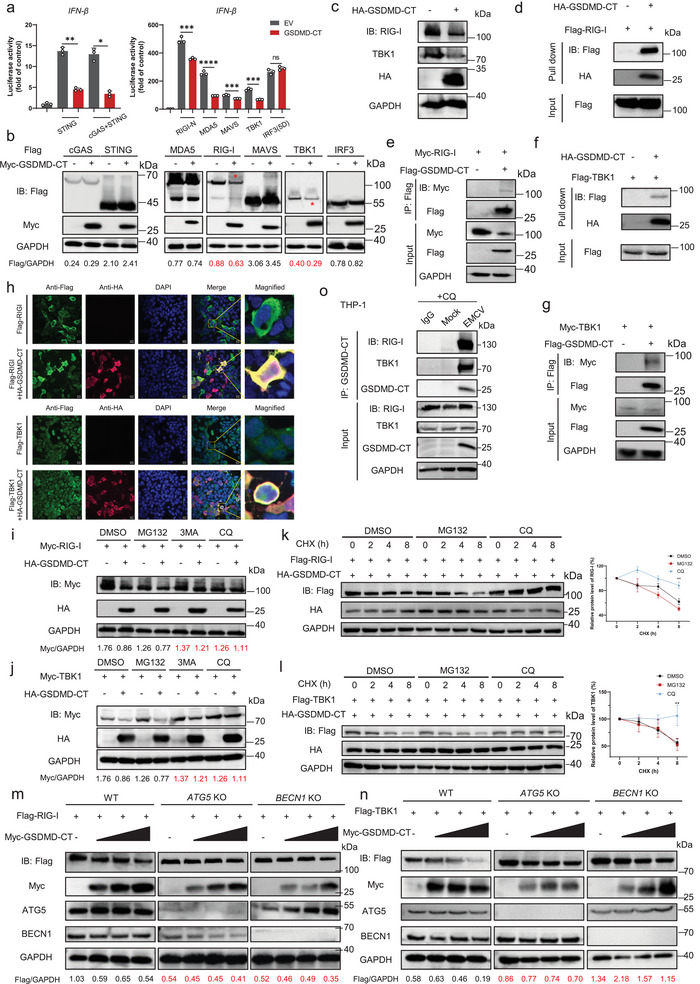
GSDMD‐CT promotes the autophagic degradation of RIG‐I and TBK1. a) Luciferase reporter assays analyzing *IFN‐β* promoter activity of HEK293T cells transfected with GSDMD‐CT or empty vector (EV) for 24 h, together with the indicated plasmids. Data are represented as mean ± SD. ^****^ *p* < 0.0001, ^***^ *p* < 0.001, ^**^ *p* < 0.01, ^*^ *p* < 0.05, NS, not significant (*p* > 0.05) (Student's *t*‐test). b) Immunoblot analysis of cells in (a). The red asterisks indicated the position of the corresponding target bands. c) HEK293T cells were transfected with EV or HA‐GSDMD‐CT for 24 h. Cell lysates were collected for immunoblot analysis with indicated antibodies. d, f) HEK293T cells were transfected with HA‐tagged Empty Vector (EV), Flag‐RIG‐I, Flag‐TBK1, and HA‐GSDMD‐CT separately for 24 h. The corresponding proteins were enriched using beads with specific antibodies. Flag‐tagged proteins were eluted from Flag beads using Flag peptide and subsequently incubated with HA‐tagged proteins immobilized on HA beads. Flag proteins were then detected using an anti‐Flag antibody. e,g) Coimmunoprecipitation and immunoblot analysis of HEK293T cells transfected with indicated plasmids. h) Immunofluorescence microscopy and nuclear staining (with the DNA‐binding dye DAPI) of HEK293T cells transfected with expression plasmids for Flag‐RIG‐I, Flag‐TBK1, and HA‐GSDMD‐CT. Scale bars, 20 µm. i,j) HEK293T cells were transfected with Myc‐RIG‐I or Myc‐TBK1, along with EV or HA‐GSDMD‐CT, followed by treatments of MG132 (10 µm), 3MA (10 mm), and CQ (40 µm) for 6 h, respectively. The cell lysates were then analyzed by immunoblot. k,l) HEK293T cells were transfected with Flag‐RIG‐I or Flag‐TBK1, along with EV or HA‐GSDMD‐CT, followed by treatments of MG132 (10 µm), and CQ (40 µm) for 6 h, as well as cycloheximide (CHX, 25 µg mL^−1^) the indicated time points, respectively. The cell lysates were then analyzed by immunoblot. Quantitative analysis of relative protein levels. Data are represented as mean ± SEM. ^**^ *p* < 0.01 (Two‐way ANOVA) m, n) WT, *ATG5* KO, and *BECN1* KO HEK293T cells were transfected with Flag‐RIG‐I or Flag‐TBK1, along with EV or HA‐GSDMD‐CT. The cell lysates were then analyzed by immunoblot. o) THP‐1 cells were treated with PMA (1 µm) for 24 h and then EMCV (MOI = 1) infection for 24 h, with CQ (40 µm) treatment for 6 h. Cell lysates were collected and immunoprecipitated with control IgG or anti‐GSDMD‐CT antibodies.

To explore the specific molecular mechanism by which GSDMD‐CT regulates the IFN‐I signaling pathway, we conducted a screen using a dual luciferase reporter assay. Our results revealed that overexpression of GSDMD‐CT markedly diminished *IFN‐β* promoter activity induced by co‐transfecting plasmids expressing STING, cGAS plus STING, RIG‐I‐N, MDA5, MAVS, and TBK1, except for IRF3 (5D) (**Figure** **4**a). Immunoblotting results showed that GSDMD‐CT significantly decreased the protein levels of RIG‐I and TBK1 (Figure 4b,c) but did not affect their mRNA levels (Figure S4a,b, Supporting Information). After conducting co‐IP assays and pull‐down experiments, we discovered that GSDMD‐CT interacted with RIG‐I and TBK1 both in vivo and in vitro (Figure 4d–g; Figure S4c,d, Supporting Information). Additionally, confocal microscopy analysis revealed the colocalization of GSDMD‐CT with RIG‐I and TBK1 in the cytoplasm (Figure 4h; Figure S4e,f, Supporting Information). Moreover, we discovered that GSDMD‐CT interacted with RIG‐I and TBK1 at multiple sites, as all their domains could interact with GSDMD‐CT (Figure S4g–j, Supporting Information).

Having observed that GSDMD‐CT promotes the degradation of RIG‐I and TBK1, our subsequent objective was to determine whether this degradation occurs via the ubiquitin‐proteasome pathway or the autolysosome pathway. Initially, HEK293T cells were co‐transfected with GSDMD‐CT and RIG‐I or TBK1, followed by treatment with the proteasome inhibitor MG132 or the autophagosome‐lysosome inhibitors 3‐methyladenine (3MA) and chloroquine (CQ). The results demonstrated that treatment with 3MA or CQ rescued the degradation of RIG‐I or TBK1 induced by GSDMD‐CT (Figure 4i,j). Subsequently, the cells were further treated with cycloheximide (CHX) to inhibit new protein synthesis, revealing that autophagosome‐lysosome inhibitor significantly prevented the degradation of RIG‐I or TBK1(Figure 4k,l). The degradation of RIG‐I or TBK1 triggered by GSDMD‐CT was almost abrogated in *ATG5*‐KO and *BECLIN1*(*BECN1*)‐KO HEK293T cells (Figure 4m,n; Figure S4k, Supporting Information). We further found that under virus infection conditions, endogenous GSDMD‐CT, cleaved from GSDMD, could interact strongly with RIG‐I and TBK1 (Figure 4o). Taken together, these results demonstrate that GSDMD‐CT specifically degrades RIG‐I and TBK1 through autophagy.

### 2.5 GSDMD‐CT Enhances the Recognition of RIG‐I by NDP52 and TBK1 by TOLLIP

Cargo receptors play a pivotal role in selective autophagy by delivering substrates to autophagosomes.^[^ ^22^, ^23^, ^24^ ^]^ Thus, we next intended to identify the potential cargo receptor responsible for the autophagic degradation of RIG‐I or TBK1 induced by GSDMD‐CT. We first performed co‐IP assays using several receptors, including p62/SQSTM1, NDP52, neighbor of BRCA1 (NBR1), TAX1BP1 (TAX), OPTN, toll interacting protein (TOLLIP), and Nip‐like protein X (Nix), to assess the interaction between GSDMD‐CT and these receptors. We found that GSDMD‐CT interacted with all the receptors above (Figure S5a, Supporting Information). RIG‐I was found to interact with NDP52, NBR1, OPTN, TOLLIP, and Nix (Figure S5b, Supporting Information), while TBK1 interacted with p62/SQSTM1, NDP52, OPTN, TOLLIP, and Nix (Figure S5c, Supporting Information). Further studies revealed that the interaction between RIG‐I and NDP52 and TOLLIP was enhanced in the presence of GSDMD‐CT (Figure S5d, Supporting Information), while the interaction between TBK1 and NDP52 was also strengthened in the presence of GSDMD‐CT (Figure S5e, Supporting Information). We subsequently knocked down NDP52 and TOLLIP expression using siRNA and observed that knocking down NDP52 rescued GSDMD‐CT‐mediated degradation of RIG‐I, while knocking down TOLLIP rescued the degradation of TBK1 induced by GSDMD‐CT (Figure S5f–i, Supporting Information). Moreover, in vitro, pull‐down experiments confirmed direct interactions between GSDMD‐CT and RIG‐I, as well as between GSDMD‐CT and TBK1 (Figure S5j,k, Supporting Information). Consistently, knocking down NDP52 and TOLLIP both rescued the inhibition of VSV‐induced *IFNB* and *Isg54* transcription (Figure S5l,m, Supporting Information). Collectively, GSDMD‐CT promotes the interaction between RIG‐I and NDP52, as well as TBK1 and TOLLIP, facilitating selective autophagic degradation.

### 2.6 GSDMD‐CT Promotes the Polyubiquitination of RIG‐I and TBK1

**Figure 5:**
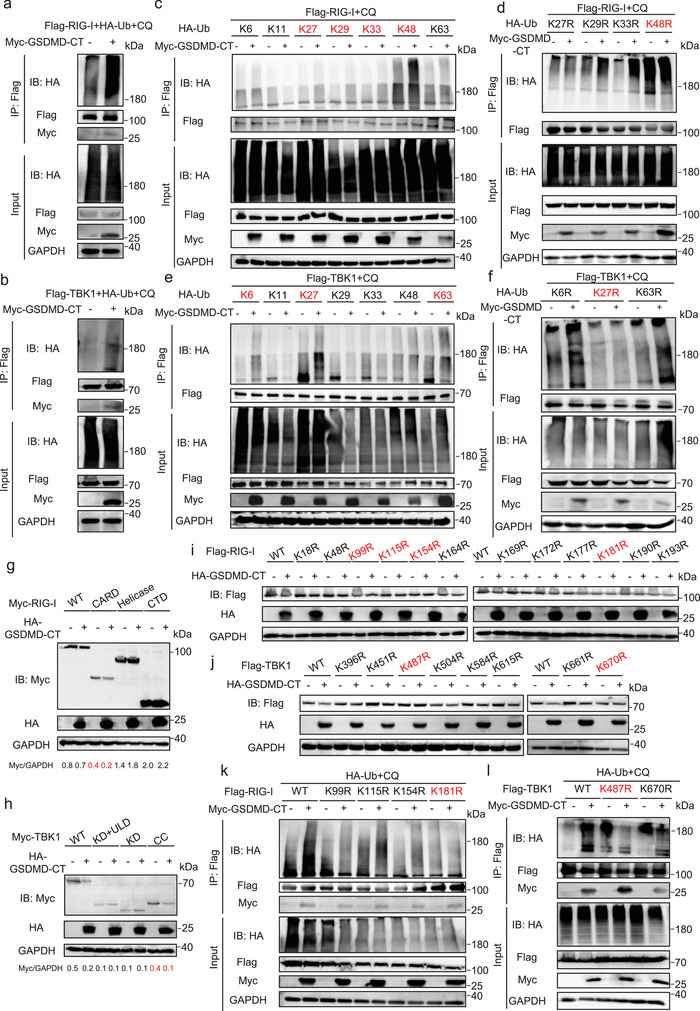
GSDMD‐CT promotes the polyubiquitination of RIG‐I and TBK1. a,b) Coimmunoprecipitation and immunoblot analysis of HEK293T cells transfected with Flag‐RIG‐I (a) or Flag‐TBK1 (b), along with the Myc‐GSDMD‐CT and HA‐ ubiquitin (Ub), in the presence of CQ (40 µm) for 6 h. c–f) Coimmunoprecipitation and immunoblot analysis of HEK293T cells transfected with Flag‐RIG‐I c,d) or Flag‐TBK1 e,f) along with the Myc‐GSDMD‐CT and indicated Ub mutants, in the presence of CQ (40 µm) for 6 h. g,h) HEK293T cells were transfected with wild‐type (WT) or deletion mutants of Myc‐RIG‐I (g) or Myc‐TBK1 (h), together with or without HA‐GSDMD‐CT for 24 h. Cell lysates were collected for immunoblot analysis. i,j) HEK293T cells were transfected with WT or mutants of Flag‐RIG‐I (i) or Flag‐TBK1 (j), together with or without HA‐GSDMD‐CT for 24 h. Cell lysates were collected for immunoblot analysis. k,l) Coimmunoprecipitation and immunoblot analysis of HEK293T cells transfected with WT or mutants of Flag‐RIG‐I (k) or Flag‐TBK1 (l), along with the Myc‐GSDMD‐CT and HA‐Ub.

Ubiquitination serves as a common signal for selective autophagy. Ubiquitin‐dependent selective autophagy receptors have the capability to bind cargo and ubiquitin simultaneously, thereby initiating pathways that lead to autophagy initiation and membrane recruitment.^[^ ^25^, ^26^ ^]^ Thus, we investigated whether GSDMD‐CT influenced the ubiquitination of RIG‐I and TBK1. We conducted co‐IP assays and observed that GSDMD‐CT distinctly promotes the ubiquitination of RIG‐I and TBK1 (**Figure** **5**a,b). Further investigation demonstrated that GSDMD‐CT enhances multiple ubiquitin linkages on RIG‐I, specifically K27‐, K29‐, K33‐, and K48‐linked ubiquitination (Figure 5c). To determine the critical ubiquitin chain type, we performed experiments using ubiquitin mutants. Mutating K27, K29, or K33 had no discernible effect on RIG‐I ubiquitination, whereas the K48R mutant completely abolished it This suggests that K48‐linked ubiquitin chains are required for GSDMD‐CT‐mediated RIG‐I ubiquitination (Figure 5d). Similar experiments were performed for TBK1, revealing that K27‐linked ubiquitination is the primary modification promoted by GSDMD‐CT (Figure 5e,f). To determine the specific sites for ubiquitination of RIG‐I and TBK1, we first tested which domain of the two proteins could be degraded by GSDMD‐CT. The immunoblotting results indicated that the CRAD domain of RIG‐I and the CC domain of TBK1 could be degraded by GSDMD‐CT, while other domains were not affected (Figure 5g,h). We subsequently employed GPS‐Uber,^[^ ^27^ ^]^ a hybrid‐learning framework for predicting general and E3‐specific lysine (K) ubiquitination sites, to identify potential lysine sites within the responsible domain. By further analyzing the sequence of human GSDMD and orthologs, we selected the following residues for RIG‐I: K18, K48, K99, K115, K154, K164, K169, K172, K177, K181, K190, and K193, and for TBK1: K396, K451, K487, K504, K584, K615, K661, and K670, to potentially serve as ubiquitination sites (Figure S6, Supporting Information). After mutating these residues to arginine (R), we found that RIG‐I‐K18R, K115R, K172R, and K181R mutants would not be degraded by GSDMD‐CT, while TBK1‐K487R and K670R would not be degraded (Figure 5i,j). We further performed co‐IP assays to confirm that GSDMD‐CT no longer facilitated the ubiquitination of RIG‐I‐K181R and TBK1‐K487R (Figure 5k,l). Our results suggest that GSDMD‐CT‐induced autophagic degradation of RIG‐I and TBK1 is dependent on K48‐linked polyubiquitination of RIG‐I at Lys181, and K27‐linked polyubiquitination of TBK1 at Lys487.

### 2.7 GSDMD‐CT Enhances the Polyubiquitination of RIG‐I and TBK1 through E3 Ubiquitin Ligase TRIM28

**Figure 6:**
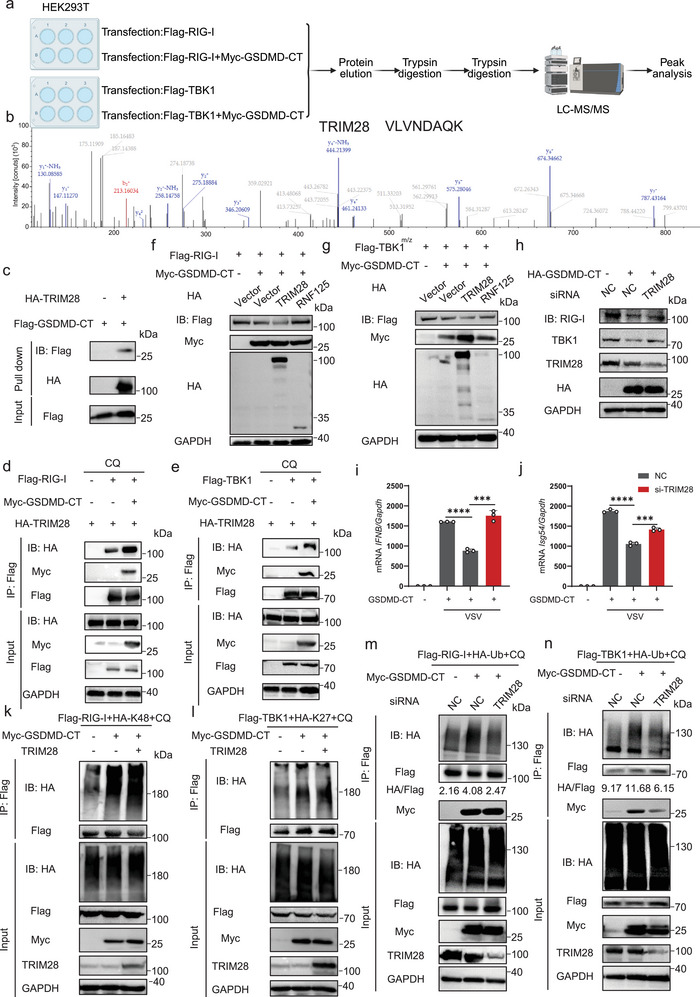
GSDMD‐CT enhances the polyubiquitination of RIG‐I and TBK1 through E3 ubiquitin ligase TRIM28. a) Schematic of the approach and strategy used to identify the specific E3 ubiquitin ligase. b) Mass spectrometry analysis. TRIM28 was identified by marker peptide “VLVNDAQK”. c) HEK293T cells were transfected with HA‐tagged Empty Vector (EV) and Flag‐GSDMD‐CT separately for 24 h. The corresponding proteins were enriched using beads with specific antibodies. Flag‐tagged proteins were eluted from Flag beads using Flag peptide and subsequently incubated with HA‐tagged proteins immobilized on HA beads. Flag proteins were then detected using an anti‐Flag antibody. d,e) Coimmunoprecipitation and immunoblot analysis of HEK293T cells transfected with HA‐TRIM28 and Flag‐ RIG‐I (d) or Flag‐TBK1 (e), along with or without Myc‐GSDMD‐CT, in the presence of CQ (40 µm) for 6 h. f,g) Immunoblot analysis of HEK293T cells transfected with Myc‐GSDMD‐CT and Flag‐ RIG‐I (f) or Flag‐TBK1 (g), along with indicated E3 ligases or empty vector. h) HEK293T cells were transfected with control siRNA or TRIM28 siRNA for 24 h, then the cells were transfected with EV or HA‐GSDMD‐CT for 24 h. Cell lysates were collected for immunoblot analysis. i,j) HEK293T cells were transfected with control siRNA or TRIM28 siRNA for 24 h, then the cells were transfected with EV or HA‐GSDMD‐CT for 24 h, followed by VSV infection for 12 h. Indicated gene expression was determined by RT–PCR. Data are represented as mean ± SD. ^****^ *p* < 0.0001, ^***^ *p* < 0.001 (Student's *t*‐test). k) Coimmunoprecipitation and immunoblot analysis of HEK293T cells transfected with HA‐K48, Myc‐GSDMD‐CT, HA‐TRIM28, and Flag‐ RIG‐I, in the presence of CQ (40 µm) for 6 h. l) Coimmunoprecipitation and immunoblot analysis of HEK293T cells transfected with HA‐K48, Myc‐GSDMD‐CT, HA‐TRIM28, and Flag‐TBK1, in the presence of CQ (40 µm) for 6 h. m,n) Coimmunoprecipitation and immunoblot analysis of HEK293T cells transfected with control siRNA or TRIM28 siRNA for 24 h, followed by transfection with Flag‐ RIG‐I (m) or Flag‐TBK1 (n) and empty vector or HA‐GSDMD‐CT for 24 h, in the presence of CQ (40 µm) for 6 h.

Since GSDMD‐CT is not an E3 ubiquitin ligase, we hypothesized that GSDMD‐CT might recruit an E3 ligase to ubiquitinate and thus degrade RIG‐I and TBK1. We performed a mass spectrometry analysis of Flag‐tagged RIG‐I or Flag‐tagged TBK1 co‐transfected with or without GSDMD‐CT (**Figure** **6**a). This analysis identified Tripartite motif‐containing 28 (TRIM28) as a specific E3 ubiquitin ligase of RIG‐I by marker peptide “VLVNDAQK” (Figure 6b). We also noticed that Ring Finger Protein 125 (RNF125) was significantly up‐regulated in Dox inducible GSDMD‐CT THP‐1 cells (Figure 3h). TRIM4, TRIM25, and TRIM32 have also been reported to play important roles in type I interferon signaling.^[^ ^28^, ^29^, ^30^ ^]^ We then investigated the interaction between these E3 ligases and GSDMD‐CT. The results indicated that all these E3 ligases could interact with GSDMD‐CT (Figure S7a–e, Supporting Information). However, only the interaction between TRIM28 and RIG‐I or TBK1 was promoted in the presence of GSDMD‐CT (Figure 6d,e; Figure S7f–i, Supporting Information). Additionally, the interactions between TRIM28 and RIG‐I, as well as TBK1, were verified (Figure S7j). We further validated the interaction between GSDMD‐CT and TRIM28 through Co‐IP with reversed tags and confocal microscopy (Figure S7k–m). The in vitro interaction between GSDMD‐CT and TRIM28 was also confirmed (Figure 6c). Overexpressing TRIM28 promotes the degradation of RIG‐I and TBK1 induced by GSDMD‐CT (Figure 6f,g), while knockdown of TRIM28 efficiently counteracted the degradation (Figure 6h). Consistently, knocking down TRIM28 potentiates the antiviral IFN‐I response induced by VSV infection (Figure 6i,j). Co‐IP assays and invitro pull‐down assays both showed that TRIM28 could promote the K48‐linked‐ubiquitination of RIG‐I and K27‐linked‐ubiquitination of TBK1 (Figure 6k,l; Figure S8a–h, Supporting Information), whereas knocking down TRIM28 inhibited GSDMD‐CT‐induced ubiquitination of RIG‐I and TBK1 (Figure 6m,n). Taken together, TRIM28 plays a critical role in the GSDMD‐CT‐induced degradation of RIG‐I and TBK1 by catalyzing the ubiquitination of RIG‐I and TBK1, thereby inhibiting IFN‐I signaling.

### 2.8 GSDMD‐CT Overexpression Increases Susceptibility to EMCV Infection In Vivo

**Figure 7:**
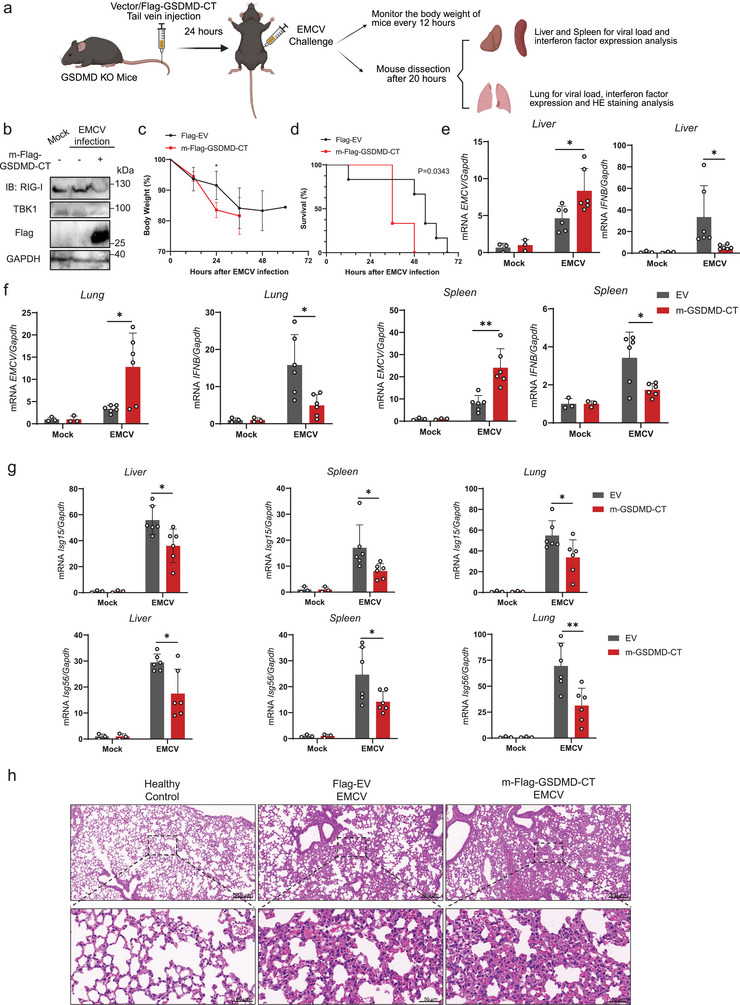
GSDMD‐CT overexpression increases susceptibility to EMCV infection in vivo. a) Schematic of the approach and strategy used to investigate the effect of GSDMD‐CT overexpression in EMCV infection. b) Indicated protein levels of the lung tissues were detected by immunoblot analysis. c) Weight of mice transfected with empty vector or Flag‐GSDMD‐CT (*n* = 6 mice per group) for 24 h, followed by intraperitoneal injection of EMCV (1 × 10^6^ PFU per mouse). ^*^ *p* < 0.05 (Student's *t*‐test). d) Survival of mice transfected with empty vector or Flag‐GSDMD‐CT (*n* = 6 mice per group) for 24 h, followed by intraperitoneal injection of EMCV (1 × 10^6^ PFU per mouse). Log‐rank (Mantel‐Cox) test. e–g) RT‐PCR analysis of *EMCV*, *IFNB*, *Isg15*, and *Isg56* mRNA levels in the liver, spleen, and lung from mice transfected with empty vector or Flag‐GSDMD‐CT, followed by treatment with phosphate‐buffered saline (PBS) or EMCV (1 × 10^6^ PFU per mouse) via intraperitoneal injection for 20 h. ^**^ *p* < 0.01, ^*^ *p* < 0.05 (Student's *t*‐test). h) Representative hematoxylin and eosin (H&E)‐stained images of lung sections from mice as described in (e‐g). Scale bars, 200 µm (top) and 50 µm (bottom).

To comprehensively investigate the physiological role of GSDMD‐CT in host defense against viral infection in vivo, we overexpressed GSDMD‐CT in GSDMD‐KO mice via tail vein injection of a plasmid encoding Flag‐tagged mouse GSDMD‐CT with an in vivo transfection reagent for 24 h, followed by EMCV infection through intraperitoneal injection (**Figure** **7**a). Overexpression of GSDMD‐CT resulted in a notable reduction in the protein levels of RIG‐I and TBK1 in the lung (Figure 7b). GSDMD‐CT overexpression accelerated the death of mice infected with EMCV, though the weight loss was not significant and may be attributed to the acute death (Figure 7c,d). Moreover, viral proliferation was significantly enhanced in the liver, spleen, and lung of mice overexpressing GSDMD‐CT, while transcription of *IFNB*, *Isg15*, and *Isg56* was inhibited (Figure 7e–g). Consistently, the severity of pulmonary inflammation was further exacerbated in GSDMD‐CT‐overexpressing mice (Figure 7h). In conclusion, our results demonstrate that GSDMD‐CT inhibits host defense against viral infection.

### 2.9 P414, Q416, and E459 Are Crucial for GSDMD‐CT in Attenuating the Type I Interferon Response

**Figure 8:**
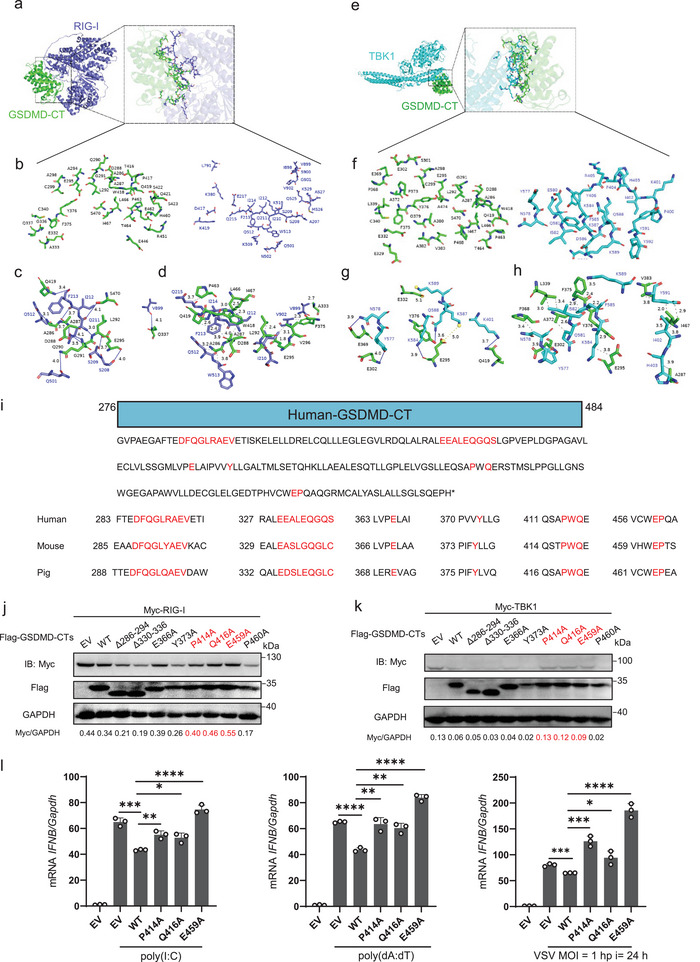
P414, Q416, and E459 are important for GSDMD‐CT to inhibit the type I interferon response. a) The conformation of the protein‐protein docking complex between mouse GSDMD‐CT (in green) and RIG‐I (in blue). PyMOL was used to analyze interfacial interactions by identifying the residues located at the protein‐protein interface. b) A distribution of the interfacial residues from (a). c) Hydrogen bonds present at the interface residues from (a). d) Hydrophobic interactions occurring at the interface residues from (a). e) The conformation of the protein‐protein docking complex between mouse GSDMD‐CT (in green) and TBK1(in cyan). PyMOL was used to analyze interfacial interactions by identifying the residues located at the protein‐protein interface. f) A distribution of the interfacial residues from (e). g) Hydrogen bonds present at the interface residues from (e). h) The salt bridges formed between GSDMD‐CT and TBK1. i) Scheme of human GSDMD‐CT protein marked with candidate sites interacted with RIG‐I or TBK1. Sequence alignments of these sites within GSDMD orthologues from different species are shown below. j,k) HEK293T cells were transfected with Myc‐RIG‐I (j) or Myc‐TBK1 (k), along with EV or Flag‐GSDMD‐CT or indicated Flag‐GSDMD‐CT mutants for 24 h. The cell lysates were then analyzed by immunoblot. l) RT‐PCR analysis of *IFNB* mRNA level in the HEK293T cells transfected with EV or Flag‐GSDMD‐CT or indicated Flag‐GSDMD‐CT mutants for 24 h, followed by poly(I:C) or poly(dA:dT) stimulation for 12 h or VSV infection for 12 h.

Considering the negative impact of GSDMD‐CT on IFN‐I signaling, identifying the specific amino acid sites that mediate this function is crucial, as it may offer avenues for controlling viral infections and developing therapeutic strategies. To investigate the binding region and interaction mode between GSDMD‐CT (PDB:6Ao3) and RIG‐I (Uniprot: Q6Q899) or TBK1 (Uniprot: Q9WUN2), we used ZDOCK3.0.2 for docking and scoring, integrating shape complementarity, electrostatic, and statistical potential terms. Protein‐protein interface residues were analyzed using pymol Interface Residue. The molecular structures of the mouse proteins were sourced from the RCSB Protein Data Bank (RCSB PDB). Scoring was based on factors such as shape complementarity, electrostatic interactions, and statistical potential terms, with higher scores suggesting stronger binding affinities. For the docking of GSDMD‐CT with RIG‐I, the highest ZDOCK score was 1271.270. The corresponding protein‐protein docking complex conformation is depicted in **Figure** **8**a, showing GSDMD‐CT in green and RIG‐I in blue. PyMOL was utilized to analyze interfacial interactions by identifying residues at the protein‐protein interface. Figure 8b displays the distribution of interfacial residues. Hydrogen bonds at the interface residues are summarized in Table S2 (Supporting Information) and illustrated in Figure 8c, while hydrophobic interactions are detailed in Table S3 (Supporting Information) and shown in Figure 8d. In the docking between GSDMD‐CT and TBK1, the highest ZDOCK score recorded was 1475.078. The corresponding protein‐protein docking complex conformation is illustrated in Figure 8e, with GSDMD depicted in green and TBK1 in cyan. PyMOL was again used to identify interface residues. Figure 8f shows their distribution. Hydrogen bonds between GSDMD‐CT and TBK1 are documented using Plip in Table S4 (Supporting Information) and Figure 8g. Hydrophobic interactions formed between GSDMD‐CT and TBK1 are shown in Table S5 (Supporting Information). Additionally, Figure 8h highlights the salt bridges formed between GSDMD‐CT and TBK1, specifically noting that E295 of GSDMD‐CT forms a salt bridge with K587 of TBK1 (distance of 5.0 Å), and E332 of GSDMD‐CT forms one with K589 of TBK1 (distance of 5.1 Å).

Through analysis of the overlapping regions of mouse GSDMD‐CT binding with RIG‐I and TBK1, we determined that GSDMD‐CT may bind with RIG‐I and TBK1 at multiple sites. Sequence comparison revealed that Asp288‐Val296 (human orthologs Asp286‐Val294), Glu332‐Cys338 (human orthologs Glu330‐Ser338), Glu369 (human orthologs Glu366), Tyr376 (human orthologs Tyr 373), Pro417 (human orthologs Pro414), Trp418 (human orthologs Trp415), Gln419 (human orthologs Gln416), Glu462 (human orthologs Glu459), Pro463 (human orthologs Pro460) are conserved in GSDMD orthologs across species (Figure 8i). We then constructed Asp286‐Val294 depletion and Glu330‐Ser338 depletion mutants, as well as single‐site Ala mutants, as mentioned previously in human GSDMD‐CT. Considering the potential of ubiquitination and acetylation to affect protein function, we also constructed Lys299 and Lys387 mutants. We first transfected GSDMD‐CT and mutants in HEK293T cells together with RIG‐I or TBK1. The results demonstrated that P414A, Q416A, and E459A mutants of GSDMD‐CT lost the ability to degrade RIG‐I or TBK1 (Figure 8j,k). We further transfected these mutants in HEK293T cells followed by poly(I:C) or poly(dA:dT) stimulation, or VSV infection, and found that all these mutants could promote the transcription of *IFNB* (Figure 8l), suggesting that P414, Q416, and E459 are crucial for GSDMD‐CT in attenuating the type I interferon response.

## 3 Discussion

**Figure 9:**
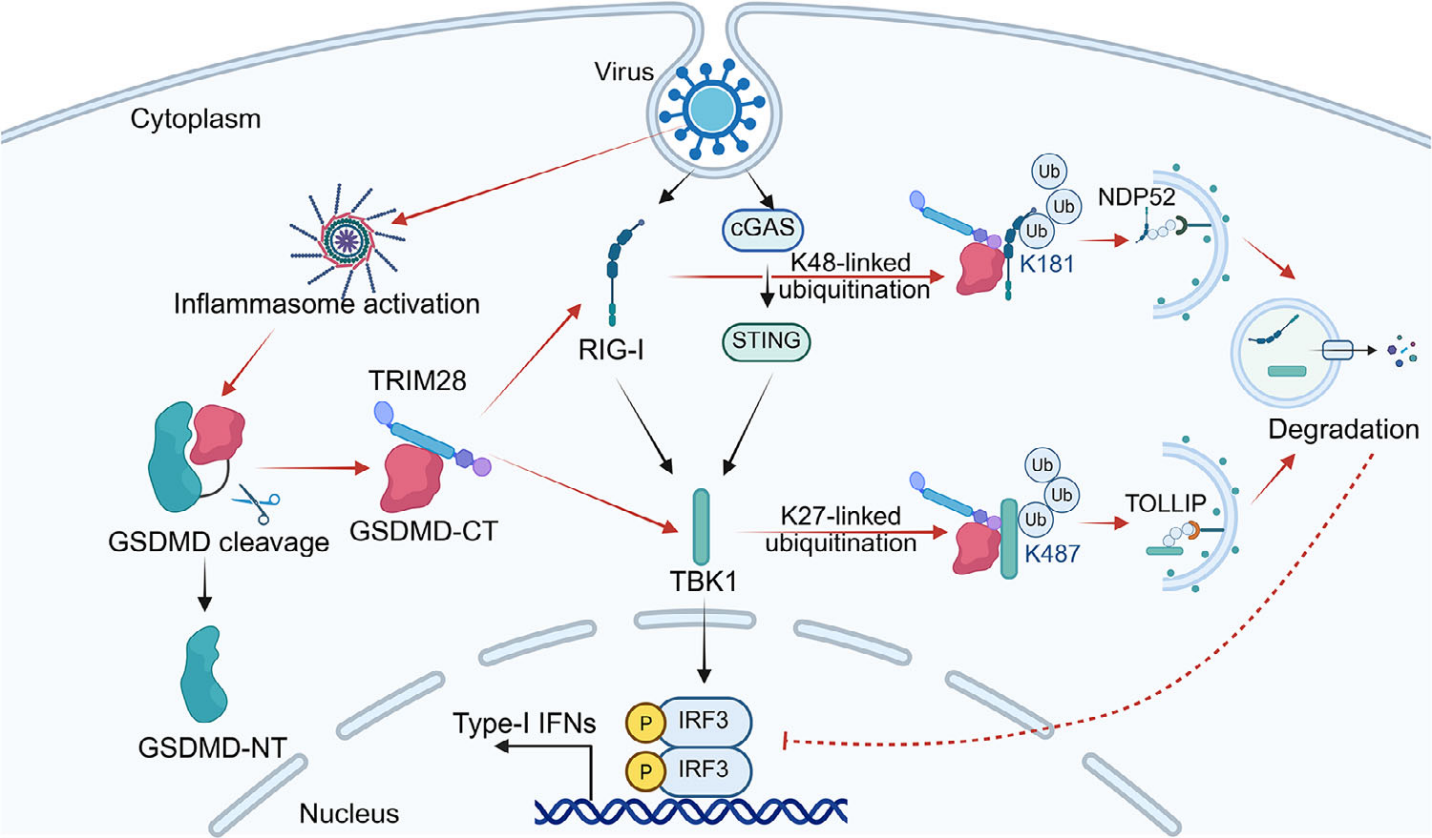
Viral infection triggers GSDMD‐CT release, which attenuates IFN‐I signaling. GSDMD‐CT targets RIG‐I and TBK1 for selective autophagy by promoting their distinct polyubiquitination by the E3 ligase TRIM28. K48‐linked polyubiquitination of RIG‐I (Lys181) engages NDP52, while K27‐linked polyubiquitination of TBK1 (Lys487) engages TOLLIP for autophagic degradation.

In the context of virus infection, inflammasome activation resembles a strategic game between the host and pathogens. Activation of inflammasomes and subsequent inflammatory responses play pivotal roles in controlling infections. Conversely, pathogens employ diverse strategies to counter host defense mechanisms. Previous work has demonstrated that various inflammasome components negatively regulate type I‐IFN induction. For instance, DNA viruses exploit Caspase‐1, activated through canonical and non‐canonical inflammasome pathways, to cleave cGAS, thus suppressing cGAS‐STING‐mediated IFN production.^[^ ^31^ ^]^ NLRC3 has been found to interact with STING and TBK1, preventing proper trafficking of STING to the perinuclear and punctated regions, thereby impeding its activation and the interaction between STING and TBK1.^[^ ^32^ ^]^ NLRC5 can interact with RIG‐I and MDA5 but not with MAVS, thereby inhibiting RLR‐mediated type I interferon responses.^[^ ^33^ ^]^ NLRP4 recruited the E3 ubiquitin ligase DTX4 to TBK1 for K48‐linked polyubiquitination at Lys670, resulting in the degradation of TBK1.^[^ ^34^ ^]^ In this study, we have made a pioneering discovery by revealing that GSDMD‐CT, which has been overlooked for a long time as research primarily focused on the fragment that drives pyroptotic activity, GSDMD‐NT, inhibits the type I interferon response through the autophagic degradation of RIG‐I and TBK1 (**Figure** **9**). Our findings contribute to the growing evidence supporting the idea that viruses activate inflammasomes to inhibit type I interferon responses, thereby offering a novel perspective on pathogen infection and immune response.

GSDMD, the most extensively studied gasdermin family member, is a key executioner of pyroptosis. Beyond its role in pyroptosis, GSDMD has been implicated in other forms of cell death, including apoptosis, necroptosis, and NETosis, and may even mediate crosstalk between these pathways.^[^ ^35^, ^36^ ^]^ Furthermore, GSDMD participates in various immune processes independent of cell death.^[^ ^37^ ^]^ GSDMD could directly target some intracellular bacteria, forming pores in their membranes and causing bacteriolysis and release of bacterial DNA, which does not robustly activate the cGAS‐STING pathway.^[^ ^38^ ^]^ During osteoclast formation, GSDMD is cleaved by Caspases‐8 and Caspase‐3 (at D276 and D88, respectively) in a RIPK1‐dependent manner, producing the p20 fragment. This GSDMD‐p20 then targets early endosomes, forms oligomers, and blocks the conversion of PI(3)P to PI(3,5)P2. This prevents excessive maturation of early endosomes into late endosomes, thus helping maintain bone mass.^[^ ^39^ ^]^ While in the large intestine, GSDMD is essential for maintaining the mucus barrier and healthy gut microbiota,^[^ ^40^ ^]^ GSDMD maintains food tolerance in small intestine enterocytes.^[^ ^41^ ^]^ A novel GSDMD‐NT‐p40 cleavage has also been observed in lung epithelial cells upon exposure to multiple allergens. This cleavage fragment is associated with the release of IL‐33 and the activation of type II immunity, rather than the typical type I immunity.^[^ ^42^ ^]^

While pyroptosis is often linked to host defense, the role of GSDMD in the type I interferon response remains understudied. Viral infections, including those by IBDV, VSV, AIV, and NDV, can activate inflammasomes like NLRP3 (leading to Caspase‐1 activation and GSDMD cleavage)^[^ ^43^ ^]^ or directly induce GSDME cleavage.^[^ ^44^ ^]^ However, the impact of GSDMD on interferon signaling appears complex. One study suggests GSDMD‐mediated potassium efflux inhibits the cGAS‐STING pathway and thus the interferon response to cytosolic DNA.^[^ ^19^ ^]^ Conversely, others report that GSDMD's pore‐forming activity enhances unconventional IFN‐β release, restricting TGEV and PDCoV infection.^[^ ^20^ ^]^ These seemingly contradictory findings highlight the need for further investigation into the precise role of GSDMD in antiviral immunity. In GSDMD‐KO mice, IAV infection resulted in reduced mRNA levels of antiviral genes compared to WT mice, while virus titers in the lungs of both WT and KO mice were similar.^[^ ^21^ ^]^ In our study, we found that GSDMD‐FL enhances IFN‐I signaling when overexpressed in HEK293T cells and, conversely, when knocked out in THP‐1 cells (Figure 2). The differences in results between our study and that of Ishita Banerjee may arise from variations in methodologies and cell types employed. Also, due to the pyroptosis induced by GSDMD‐NT, which may involve complex mechanisms and multiple signaling pathways, we have not thoroughly investigated the specific regulatory mechanism of GSDMD‐NT in type I interferon response, which still needs to be further studied.

There is also a distinct cellular state called “hyperactivation”, where immune cells release pro‐inflammatory cytokines like interleukin‐1β while remaining viable.^[^ ^45^ ^]^ According to Evavold's research, GSDMD exhibits dual functions corresponding to two different cell‐fate decisions. The first function involves executing pyroptotic cell death, while the second mediates cell hyperactivation. The pyroptotic pathway generates an intense localized inflammatory response at infection sites, though this comes at the cost of cellular death, preventing further immunomodulatory functions of the affected cells. In contrast, cell hyperactivation allows for continued immune cell function but may result in comparatively lower IL‐1 production in infected tissues than what occurs during pyroptosis. This trade‐off between the two cell‐fate decisions highlights the complex balance between inflammatory response magnitude and sustained immune cell functionality.^[^ ^46^ ^]^ We used the osmoprotectant glycine to prevent pyroptosis‐related membrane rupture, thereby creating a “hyperactivation” scenario. Under these conditions, we found that GSDMD deficiency still notably promoted the transcription of *IFNB* and *Isg54*, suggesting that this phenomenon is independent of cell lysis.

Selective autophagy degradation has been reported to be important in regulating the type I IFN signaling pathway. Tetherin recruits E3 ubiquitin ligase MARCH 8 to promote K27‐linked ubiquitination of MAVS, which serves as a recognition signal for NDP52‐dependent autophagic degradation.^[^ ^47^ ^]^ Leucine‐rich repeat‐containing protein 25 (LRRC25) specifically binds to Isg15‐associated RIG‐I to promote the interaction between RIG‐I and the autophagic cargo receptor p62, mediating RIG‐I degradation via selective autophagy.^[^ ^48^ ^]^ NEDD4 directly catalyzes the K27‐linked polyubiquitination of TBK1, serving as a recognition signal for NDP52‐mediated selective autophagic degradation,^[^ ^49^ ^]^ while VANGL2 promotes the selective autophagic degradation of TBK1 via K48‐linked polyubiquitination by the E3 ligase TRIP, which serves as a recognition signal for the cargo receptor OPTN.^[^ ^50^ ^]^ Similarly, K48‐ubiquitinated cGAS is targeted for autophagy by p62,^[^ ^51^ ^]^ and K48‐ubiquitinated ACE2 is degraded via a TOLLIP‐dependent selective autophagy pathway.^[^ ^52^ ^]^

Our findings indicate that GSDMD‐CT recruits TRIM28 to promote K48‐linked polyubiquitination of RIG‐I at Lys181 and K27‐linked polyubiquitination of TBK1 at Lys487, serving as recognition signals for the cargo receptors NDP52 and TOLLIP, respectively.

Investigating the crucial amino acid sites of GSDMD‐CT that affect its inhibitory function on the type I IFN signaling pathway offers the potential for controlling viral infections. Interestingly, we found that the P414A, Q416A, and E459A mutants exhibited a function opposite to that of GSDMD‐CT. The detailed mechanisms by which these mutants affect the function of GSDMD‐CT need to be further explored.

There is concern that while pyroptosis is a relatively rapid process, the regulation of IFN‐I signaling by GSDMD‐CT might require more time to induce the expression of IFN‐I in pyroptotic cells. Although pyroptosis is typically characterized as a swift lytic cell death, it may not always follow an “all‐or‐nothing” pattern. Evidence indicates that cells can experience partial or temporarily delayed pyroptotic events, allowing adequate time for the initiation of transcriptional responses. Reports suggest that gasdermin‐derived pores can form in a sub‐lytic manner, producing pores large enough to permit the efflux of certain cytosolic components but not immediate cell lysis.^[^ ^8^ ^]^ During this period, cells may remain transiently viable, facilitating active transcription and translation of immune‐related genes, such as IFN‐I. Furthermore, not all pyroptotic cells die at the same speed.^[^ ^53^ ^]^ There is variability among cells in the kinetics of gasdermin pore formation and subsequent lysis. Some cells may linger long enough to initiate transcriptional programs for IFN‐I. Even a fraction of cells that survive a few hours longer could collectively produce clinically relevant levels of IFN‐I. Even if a pyroptotic cell eventually ruptures, it might release GSDMD‐CT or other signaling intermediates that neighboring cells can absorb or respond to. Additionally, it has been reported that GSDMD‐NT could serve as a negative feedback mechanism to inhibit inflammasome‐mediated activation of Caspase‐1/11 by directly binding to them.^[^ ^10^ ^]^ Taken together, these factors suggest that GSDMD‐CT might have biological functions that become evident on the timescale required for IFN‐I induction.

In conclusion, our results find that full‐length GSDMD (GSDMD‐FL) enhances the IFN‐I signaling, while the C‐terminal fragment of GSDMD (GSDMD‐CT) inhibits it. Our investigations highlight the biological function of GSDMD‐CT and provide insight into potential strategies for viruses to activate pyroptosis and hijack host immune response components, thereby shedding light on virus infection control.

## 4 Experimental Section

### Cell Culture and Treatment

HEK293T, Vero, THP‐1, and GSDMD‐KO THP‐1 cells were maintained in the laboratory. All cells were cultured at 37 °C in 95% air and 5% CO_2_. HEK293T, A549, and Vero cells were cultured in Dulbecco's modified Eagle's medium (DMEM) with 10% FBS and 1% penicillin‐streptomycin. Human monocyte cell line THP‐1 cells and M‐CSF induced BMDM cells were cultured in RPMI 1640 medium with added 10% FBS and 1% penicillin‐streptomycin. Cells were transfected with Lipofectamine 2000 (Invitrogen) or Lipo8000 (Beyotime) according to the manufacturer's instructions.

### Mouse Models

Animal experiments were conducted following guidelines for experimental animals’ welfare and ethics. All animal experiments were performed in specific pathogen‐free levels using 6‐ to 8‐week‐old mice. GSDMD KO mice were obtained from GemPharmatech. All animal studies were reviewed and approved by the Laboratory Animal Center of Zhejiang University (ZJU20240049). For in vivo viral infection, 6‐ to 8‐week‐old mice were intravenously injected with 1 × 10^4^ or 1 × 10^6^ PFU EMCV. For in vivo transfection, Flag‐mouse‐GSDMD‐CT was injected into the tail veins of mice (40 µg mouse^−1^) in the presence of in vivo DNA transfection reagent (Entranster‐in vivo; Engreen) for 24 h, according to the manufacturer's protocols. In short, the plasmid was diluted to 1 µg µL^−1^ in advance. Next, prepare the nucleic acid dilution solution (50 µL plasmid + 50 µL 10% glucose) and the transfection dilution solution (100 µL transfection reagent). Allow both solutions to sit for 10 min, mix thoroughly, and let them sit for an additional 10 min before performing tail vein injection into the mouse.

### Cell Treatment

To induce inflammasome activation, 5 × 10^5^ THP‐1 cells were plated in a 24‐well plate overnight with 1 µm PMA, and then the medium was changed the next morning. To activate the canonical NLRP3 inflammasome, cells were primed for 4 h with 500 ng mL^−1^ LPS and then stimulated for 1 h using 10 µm Nigericin. For protein degradation inhibition assays in HEK293T cells, 3‐MA (1 mm) (MCE) or CQ (40 µm) (Sigma) was used to inhibit autolysosome‐ or lysosome‐mediated protein degradation. MG132(10 µm) (APEbio) was used to inhibit proteasome‐mediated protein degradation.

### Preparation of mBMDMs

Bone marrow cells were isolated from mouse tibias and femurs. Bone marrow cells were cultured for 7 days in a medium containing mouse M‐CSF (Peprotech, 315‐02, 40 ng/mL) to obtain mBMDMs.

### Virus Infection

Viruses were titrated on Vero cells. Virus titers were measured by means of 50% of the tissue culture's infectious dose (TCID_50_). For in vitro viral infection, cells were seeded into 24‐well plates before infection and were infected with viruses at the indicated multiplicity of infection (MOI). Cell media were removed before virus inoculation. After incubation at 37 °C for 2 h, the cell culture medium was removed, the cells were washed with PBS, and fresh medium was added. For in vivo viral infection, 6‐ to 8‐week‐old mice were intravenously injected with 1 × 10^4^ or 1 × 10^6^ PFU EMCV.

### Luciferase and Reporter Assays

A total of 1 × 10^5^ HEK293T cells were plated in 24‐well plates overnight and transfected with plasmids encoding the *IFN‐β* luciferase reporter (Firefly luciferase) and pRL‐TK (Renilla luciferase), together with different plasmids as follows: Flag‐cGAS, Flag‐STING, Flag‐RIG‐I‐N, Flag‐MAVS, Flag‐TBK1 and Flag‐IRF3‐5D, and Myc‐GSDMD‐CT or empty vector for 24 h. Cells were then treated with or without VSV (MOI = 1), HSV (MOI = 1), poly(I:C) (1 µg mL^−1^), poly(dA:dT) (500 ng mL^−1^) for 12 h. Samples were collected and luciferase activity was measured using the Dual‐Luciferase Reporter Assay Kit (Beyotime Biotechnology). The activity of firefly luciferase was normalized by that of Renilla luciferase to obtain relative luciferase activity.

### Immunoblotting

Cells were harvested and lysed in RIPA lysis buffer (Beyotime Biotechnology) and added with 1% PMSF (Beyotime Biotechnology). Proteins were separated on the 10% SDS‐PAGE gel (Hangzhou Fudebio) and transferred onto the PVDF membranes (Bio‐rad). Membranes were blocked in the blocking buffer (Beyotime Biotechnology), followed by staining with primary antibodies and positioning with secondary antibodies. Chemiluminescent signals were captured by an ECL chemiluminescence imaging analysis system (Clinx Science Instruments).

### Co‐Immunoprecipitation

Cells were lysed in IP lysis buffer and added with PMSF. The supernatants were then incubated with anti‐Flag binding beads (Sigma, M8823) at 4 °C overnight. The immunocomplexes were washed and then subjected to immunoblotting analysis.

### In Vitro Pull‐Down

Plasmids were individually transfected into HEK293T cells and incubated for 24 h. The cells were then lysed in IP lysis buffer supplemented with PMSF. The supernatants were collected and incubated overnight at 4 °C with beads coated with specific antibodies. Flag‐tagged proteins were eluted from the Flag beads using Flag peptide (Sigma, SAE0194) at a concentration of 100 µg mL^−1^ for 2 h at 4 °C. This elution was followed by incubation with HA‐tagged proteins immobilized on HA beads at 4 °C overnight. Finally, the HA beads were washed and subjected to immunoblotting analysis.

### Confocal Immunofluorescence Assay

HEK293T cells were seeded on coverslips in 24‐well plates. After being transfected for 24 h, cells were fixed with Immunol Staining Fix Solution (Beyotime). Cells were incubated with primary antibodies overnight at 4 °C after being permeabilized and blocked. Alex Fluor 488/555/647‐conjugated secondary antibody was incubated for 1 h. Nuclei were stained with DAPI. Confocal micrographs were imaged using a laser confocal microscope (Olympus).

### LDH Assay

The supernatants of cells were collected and then applied to the cytotoxicity test using CytoTox 96 Reagent (Promega) according to the manufacturer's manual. OD values were collected at 492 nm on an enzyme marker (Thermo Scientific).

### ELISA

Supernatants from stimulated cells and mice serum were applied to the detection of IFN‐β according to the manufacturer's instructions. Each trial group was conducted independently three times.

### LC‐MS/MS Analysis

Immunoprecipitates of Flag‐tagged RIG‐I or TBK1, as well as Myc‐tagged GSDMD‐CT, were prepared from whole‐cell lysates or gel‐filtrated fractions. These samples were resolved on SDS‐PAGE gels, followed by the excision of protein bands. Subsequently, the excised bands underwent trypsin digestion before being subjected to LC‐MS/MS analysis. Mass spectra from Swissprot_Human served as the standard reference. Cleavage was achieved using Trypsin/P. The captured MS data were analyzed by Novogene Co. Ltd.

### Total RNA Extraction and Reverse Transcription

Cells were lysed by adding an RNA‐easy Isolation Reagent (Vazyme Biotechnology, R711) to obtain a lysis solution containing the cells. For RNA extraction from mouse organs, the AFTSpin Tissue/Cell Fast RNA Extraction Kit for Animals (ABclonal, PK30120) was used according to the manufacturer's instructions. Subsequently, the extracted RNA was reverse transcribed to cDNA using the HiScript III RT SuperMix for qPCR (+gDNA wiper) (Vazyme Biotechnology, R323).

### Quantitative Real‐Time Polymerase Chain Reaction (qRT‐PCR)

qRT‐PCR was performed with ChamQ Universal SYBR qPCR Master Mix (Vazyme Biotechnology, Q711) according to the manufacturer's requirements. Primers demanded in this analysis were listed in Tables S6 and S8 (Supporting Information).

### Library Preparation and RNA‐seq

Total RNA extracted from GSDMD‐CT inducible THP‐1 cells with indicated treatment was subjected to RNA‐seq. Library preparation and RNA‐seq were conducted by Novogene Co. Ltd.

### Construction of GSDMD‐WT/GSDMD‐D275A/GSDMD‐CT Inducible Cell Lines

The lentivirus was produced by transfection of the pLVX‐TRE3G‐h‐GSDMD‐WT/pLVX‐TRE3G‐h‐GSDMD‐D275A/pLVX‐TRE3G‐h‐GSDMD‐CT or control vector into HEK293T cells using Lipo8000 with pMD2G and PSPAX2. Supernatants were collected after 48 h incubation, filtered through a 0.45 µm filter, and subsequently used to infect GSDMD‐KO THP‐1 cells with polybrene (8 µg mL^−1^). The infected cells were spun at 300 × g for 1 h, and then 1 mL of fresh media was added. After infection for 2 days, cells were treated with 1 µg mL^−1^ puromycin and 0.8 µg mL^−1^ G418 for 72 h. Then the cells were cultured with 0.5 µg mL^−1^ puromycin and 0.4 µg mL^−1^ G418.

### RNA Interference

SiRNAs (Genepharma) specific for NDP52, TOLLIP, and TRIM28 were transfected into HEK293T cells using the GP‐transfect‐Mate according to the manufacturer's instructions. SiRNA sequences are shown in Table S9 (Supporting Information).

### In Vivo Transfection

Flag‐mouse‐GSDMD‐CT was diluted to 1 µg µL^−1^ in advance. Next, prepare the nucleic acid dilution solution (50 µL plasmid + 50 µL 10% glucose) and the transfection dilution solution (100 µL transfection reagent, Entranster‐in vivo). Allow both solutions to sit for 10 min, mix thoroughly, and let them sit for an additional 10 min before performing tail vein injection into the mouse. The mice were intravenously injected with EMCV 24 h after transfection.

### In Vitro Ubiquitination Assay

HEK293T cells were co‐transfected with Flag‐RIG‐I/TBK1 and Myc‐Ub. The cells were then lysed in IP lysis buffer supplemented with PMSF. The supernatants were collected and incubated overnight at 4 °C with beads coated with specific antibodies. Flag‐tagged proteins were eluted from the Flag beads using Flag peptide (Sigma, SAE0194) at a concentration of 100 µg mL^−1^ for 2 h at 4 °C. For the subsequent ubiquitination assay, the eluted Flag‐RIG‐I or Flag‐TBK1 was incubated with HA‐tagged proteins immobilized on HA beads in ubiquitination reaction buffer (50 mm Tris–HCl [pH 7.4], 150 mm NaCl, 5 mm MgCl2, and 10 mm DTT) for reaction at 37 °C for 2 h. The ubiquitination levels of immunoprecipitated RIG‐I or TBK1 were analyzed by Western blotting.

### Data Preprocessing and DEGs Screening

HISAT2 was used to align reads to the reference genome (GRCh37).^[^ ^54^ ^]^ Samtools was applied to convert sequence alignment/map (.SAM) format files into binary alignments/maps (.BAM) format,^[^ ^55^ ^]^ and featureCounts was used for quantifying gene expression.^[^ ^56^ ^]^ Only uniquely mapped reads were used for expression quantification. According to the workflow, DEGs were screened using the “DESeq2” package in R software (version 4.1.2) with the cutoff |Log2 fold change| > 1 and adjusted *p*‐value < 0.05.

### Structural Modeling

To study the binding regions and interaction patterns of m‐GSDMD‐CT with RIG‐I and TBK1, ZDOCK3.0.2 were utilized for docking and scoring.^[^ ^57^, ^58^ ^]^ The scoring algorithm integrates shape complementarity, electrostatics, and statistical potential terms, where higher scores indicate stronger binding affinity. The molecular docking simulation was conducted by Phadcalc (www. phadcalc.com).

### Statistical Analysis

All experiments were performed independently at least three times. Data were presented as the mean ± standard deviation (SD), analyzed, and used for statistical graphing by GraphPad Prism 8, the significance of differences was determined by Student's *t*‐test. The significance of differences ranked as: ^****^stands for *p* < 0.0001, ^***^stands for *p* < 0.001, ^**^ stands for *p* < 0.01, ^*^ stands for *p* < 0.05 and ns stands for non‐significant difference.

## Conflict of Interest

The authors declare no conflict of interest.

## Author Contributions

F.S. and W.X. conceived conceptualization. F.S. and W.X. performed methodology. W.X., S.H., W.S., J.X., S.L., Z.Y., D.L., Q. J., Y.W., Z.Z., Q.L., Y.G., Y.L., X.L., N.C., and X.F. performed investigation. F.S. and W.X. wrote the original draft. F.S., Y.Y., and W.X. performed writing –review & editing. F.S. acquired funding acquisition. F.S. and Y.Y. acquired resources. F.S. supervised the experiment.

## Supporting information



Supporting Information

Supplemental Table 1

## Data Availability

The data that support the findings of this study are available from the corresponding author upon reasonable request.
