# Supplementary material for: The Neglected Role of GSDMD C‐Terminal in Counteracting Type I Interferon Signaling
Source: Adv Sci (Weinh). 2025 Jun 20;12(33):e05255. doi: 10.1002/advs.202505255 (PMC12412611; doi:10.1002/advs.202505255)
Supplement: Supplementary file 1 — Supporting Information [file ADVS-12-e05255-s002.pdf]

ADVANCED SCIENCE
Open Access

Supporting Information

for *Adv. Sci.*, DOI 10.1002/advs.202505255

The Neglected Role of GSDMD C-Terminal in Counteracting Type I Interferon Signaling

Weilv Xu, Suhui He, Wen Shi, Jinxia Xu, Shiyang Liu, Zexu Yu, Danyue Li, Qiao Jin, Yumeng Wang, Zian Zhang, Qian Lv, Yuanxiang Ge, Yunjie Li, Xinyue Li, Nan Chen, Xinyu Fu, Yang Yang and Fushan Shi**

Supporting Information

GSDMD C-Terminal Fragment Initiates Autophagic Degradation of RIG-I/TBK1 and Suppresses the Innate Immune Response during Viral Infection

Weilv Xu, Suhui He, Wen Shi, Jinxia Xu, Shiyang Liu, Zexu Yu, Danyue Li, Qiao Jin, Yumeng Wang, Zian Zhang, Qian Lv, Yuanxiang Ge, Yunjie Li, Xinyue Li, Nan Chen, Xinyu Fu, Yang Yang, Fushan Shi**

Inventory of Supporting Information

1. Supplementary Figures

Figure S1. Identification of GSDMD-deficient mice.

Figure S2. GSDMD-full length promotes IFN-I immune response during viral infection.

Figure S3. GSDMD-CT suppresses the activation of IFN-I immune signaling.

Figure S4. GSDMD-CT interacts with RIG-I and TBK1 in multiple sites and enhances the autophagic degradation of RIG-I and TBK1.

Figure S5. GSDMD-CT facilitates the recognition of RIG-I by NDP52 and TBK1 by TOLLIP.

Figure S6. Candidate ubiquitination sites of RIG-I and TBK1.

Figure S7. GSDMD-CT faciliates the ubiquitination of RIG-I and TBK1 through the E3 ubiquitin ligase TRIM28.

Figure S8. TRIM28 faciliates the ubiquitination of RIG-I and TBK1.

2. Supplementary Tables

Table S1. DEGs list.

Table S2. Residues and bond lengths involved in hydrogen bond formation for GSDMD-CT and RIG-I.

Table S3. Residues and distances involved in hydrophobic interactions for GSDMD-CT and RIG-I.

Table S4. Residues and bond lengths involved in hydrogen bond formation for GSDMD-CT and TBK1.

Table S5. Hydrophobic interactions formed between GSDMD-CT and TBK1.

Table S6. Primers used in this study for qPCR in human.

Table S7. Primers used in this study for qPCR in mouse.

Table S8. Primers used in this study for virus detection.

Table S9. siRNA sequences.

Table S10. Key Resource.

Table S11. All identified peptides by MS.

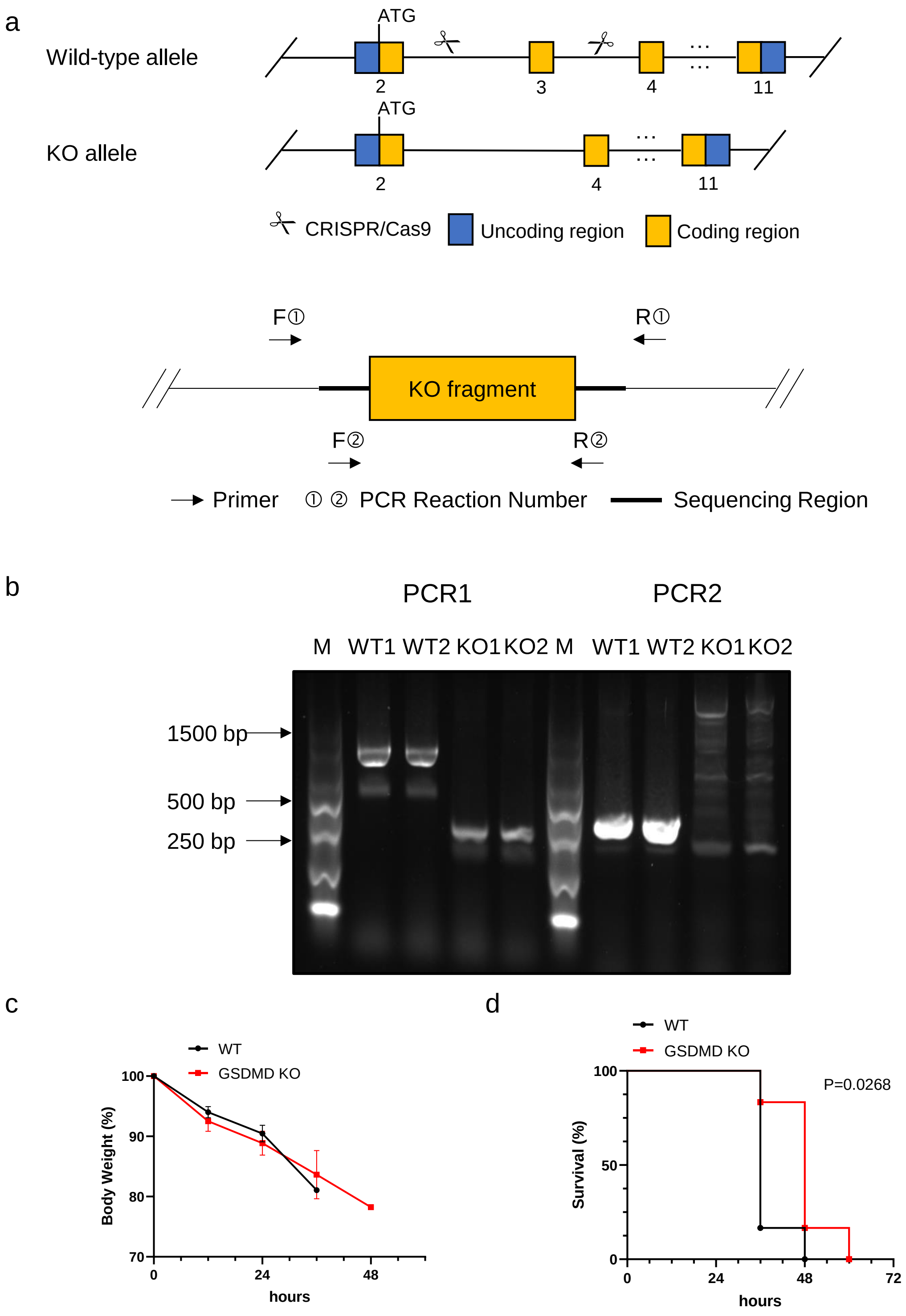

Figure S1. Identification of GSDMD-deficient mice, related to Figure 1.

a Schematic of the strategy of genotyping. **b** gel image of PCR. **c**, **d** Weight (**c**) and survival (**d**) of WT and GSDMD-deficient mice (n = 6 mice per group) after intraperitoneal injection of EMCV (1×10^6 PFU per mouse).

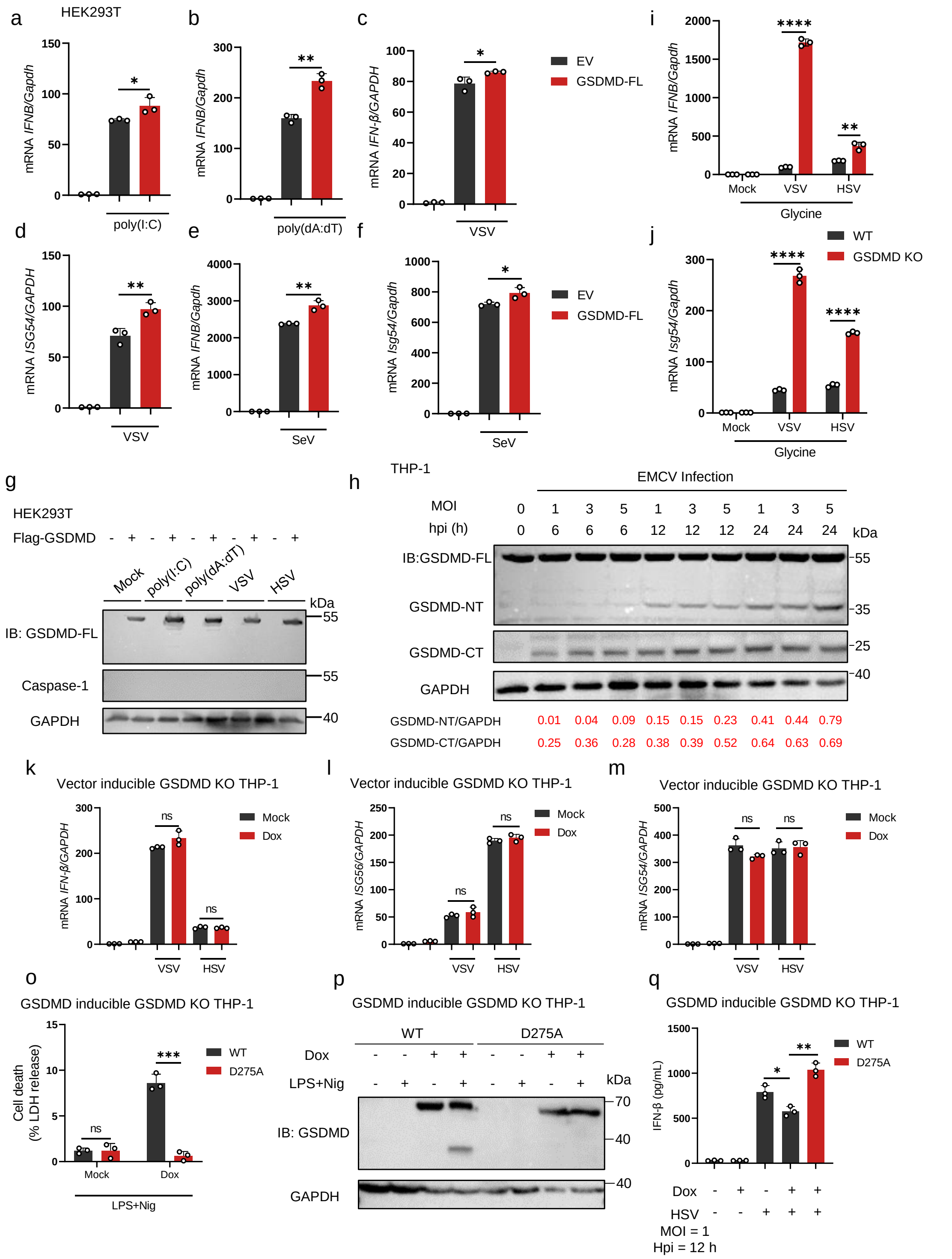

Figure S2. GSDMD-full length promotes IFN-I immune response during viral infection, related to Figure 2.

a-f RT-PCR analysis of *Ifnb* and *ISG54* mRNA levels in the HEK293T cells transfected with GSDMD-FL or EV for 24 h, followed by treatment with or without poly(I:C), poly(dA:dT), VSV (MOI = 0.1) or SeV (MOI = 0.1) for 12 h. Data are represented as mean ± SD. $**p < 0.01$, $*p < 0.05$ (Student's t test). **g** Immunoblot

analysis of HEK293T cells transfected with Flag-GSDMD-FL or EV for 24 h, followed by indicated stimulation or infection for 12 h. **h** Immunoblot analysis of THP-1 cells treated with PMA (1 μM) for 24 h and then EMCV infection for indicated times with indicated MOI. **i**, **j**, PMA-differentiated WT or GSDMD-knockout (GSDMD-KO) THP-1 cells were treated with VSV (MOI = 1) or HSV (MOI = 1) for 12 h in the presence of 5 mM glycine. *IFNB* or *Isg54* mRNA level was detected by RT-PCR analysis. Data are represented as mean ± SD. ****$p < 0.0001$, **$p < 0.01$ (Student's t test). **k-m** RT-PCR analysis of *IFNB*, *Isg54* and *Isg56* mRNA levels in Vetor-inducible GSDMD-KO THP-1 cells treated with Dox (500 ng/mL) for 72 h, followed by PMA (1 μM) treatment for 24 h and then VSV (MOI = 1) or HSV (MOI = 1) infection for 12 h. Data are represented as mean ± SD. NS, not significant ($p > 0.05$) (Student's t test). **o**, **p** GSDMD-WT-inducible GSDMD-KO THP-1 cells or GSDMD-D275A-inducible GSDMD-KO THP-1 cells were treated with Dox (500 ng/mL) for 72 h, followed by PMA (1 μM) treatment for 24 h. After differentiation, cells were primed with LPS (500 ng/mL) for 4 h and then treated with Nigericin (10 μM) for 1 h. Cell supernatants were collected for lactate dehydrogenase (LDH) release assay (**o**). Cell lysates were collected for immunoblot analysis with indicated antibodies (**p**). Data are represented as mean ± SD. ***$p < 0.001$, NS, not significant ($p > 0.05$) (Student's t test). **q** GSDMD-WT-inducible GSDMD-KO THP-1 cells were treated with or without Dox (500 ng/mL) for 72 h, followed by PMA (1 μM) treatment for 24 h. After differentiation, cells were infected with HSV (MOI = 1) for 12 h. Cell supernatants were collected for IFN-β release ELISA assay. Data are represented as mean ± SD. **$p < 0.01$, *$p < 0.05$ (Student's t test).

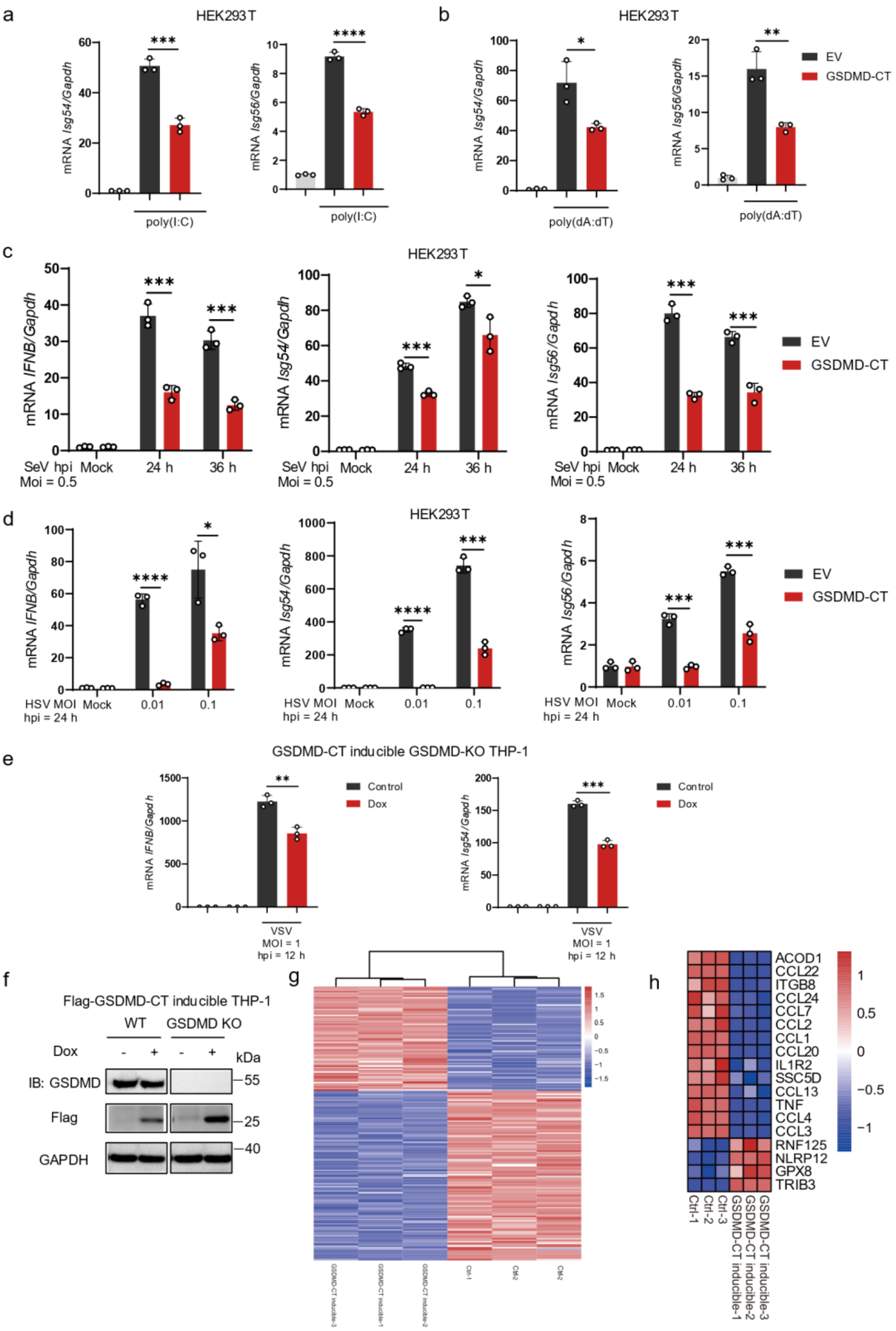

Figure S3. GSDMD-CT suppresses the activation of IFN-I immune signaling, related to Figure 3.

a, **b** RT-PCR analysis of *Isg54* and *Isg56* mRNA levels in the HEK293T cells transfected with GSDMD-CT or EV for 24 h, followed by treatment with or without

poly(I:C) or poly(dA:dT) for 12 h. Data are represented as mean ± SD. ****p < 0.0001, ***p < 0.001, **p < 0.01, *p < 0.05 (Student's t test). **c** RT-PCR analysis of indicated mRNA levels in the HEK293T cells transfected with GSDMD-CT or EV for 24 h, followed by SeV infection (MOI = 0.5) for indicated time. Data are represented as mean ± SD. ***p < 0.001, *p < 0.05 (Student's t test). **d** RT-PCR analysis of indicated mRNA levels in the HEK293T cells transfected with GSDMD-CT or EV for 24 h, followed by HSV infection at the indicated MOI for 24 h. Data are represented as mean ± SD. ****p < 0.0001, ***p < 0.001, *p < 0.05 (Student's t test). **e** RT-PCR analysis of indicated mRNA levels in Flag-GSDMD-CT inducible THP-1 cells treated with Dox (500 ng/mL) for 72 h, followed by PMA (1 μM) treatment for 24 h and then VSV (MOI = 1) infection for 12 h. Data are represented as mean ± SD. ***p < 0.001, **p < 0.01 (Student's t test). **f** Immunoblot analysis of GSDMD-CT-inducible THP-1 cells treated with or without Dox (500 ng/mL) for 72 h. **g** Heatmap of all DEGs (p-values < 0.05 and |Log2 fold change| > 1) identified. Red dots denote upregulated DEGs, while blue dots represent downregulated DEGs. **h** Heatmap showing the alterations in the expression of specified genes in control and stimulation groups.

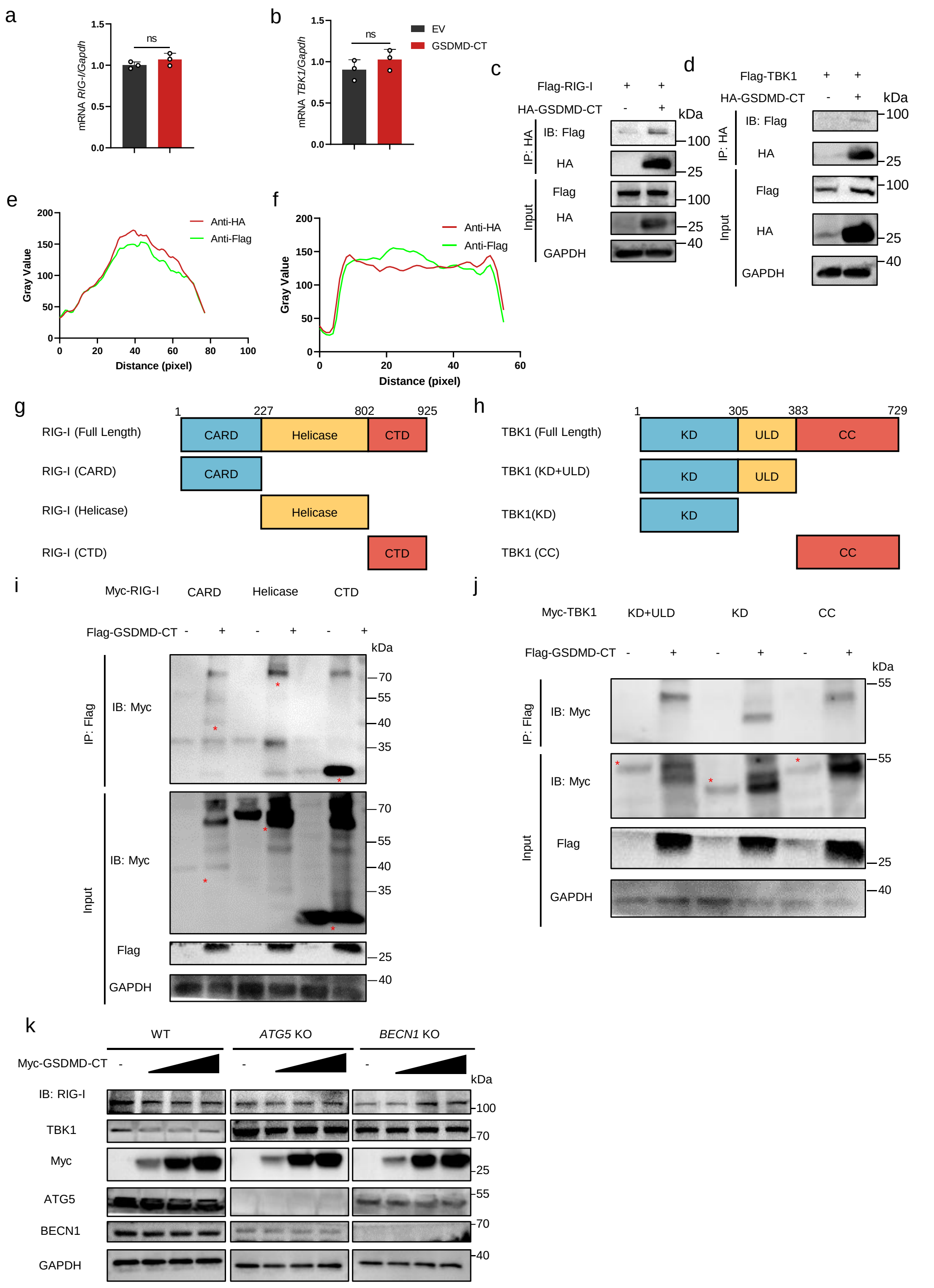

Figure S4. GSDMD-CT interacts with RIG-I and TBK1 in multiple sites and enhances the autophagic degradation of RIG-I and TBK1, related to Figure 4.

a, **b** RT-PCR analysis of indicated mRNA levels in the HEK293T cells transfected with GSDMD-CT or EV for 24 h. NS, not significant ($p > 0.05$) (Student's t test). **c**, **d** Coimmunoprecipitation and immunoblot analysis of HEK293T cells transfected with

indicated plasmids. **e**, **f** Colocalization analysis of fluorescent signals in Fig. 4h. The Gray Value represents the intensity of the fluorescent signals, while the Distance indicates the spatial separation between the fluorescently labeled structures. **g**, **h** Schematic representation of RIG-I (**g**) and TBK1 (**h**) and their mutants. **i**, **j**, Coimmunoprecipitation and immunoblot analysis of HEK293T cells transfected with Flag-GSDMD-CT and Myc-RIG-I (**i**), Myc-TBK1 (**j**) or their mutants. **k** WT, *ATG5* KO and *BECN1* KO HEK293T cells were transfected with EV or HA-GSDMD-CT. The cell lysates were then analyzed by immunoblot.

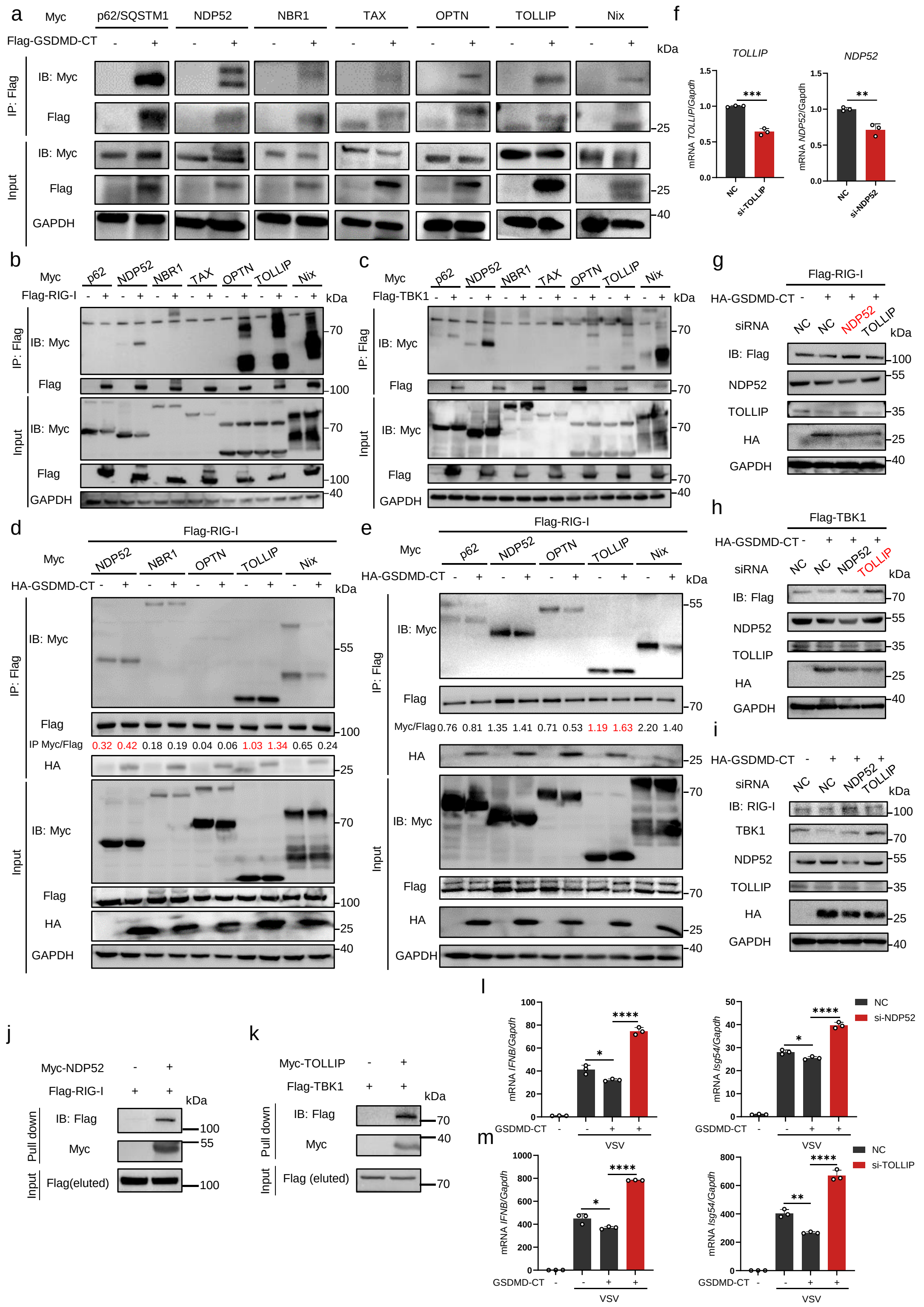

Figure S5. GSDMD-CT facilitates the recognition of RIG-I by NDP52 and TBK1 by TOLLIP.

a Coimmunoprecipitation and immunoblot analysis of HEK293T cells transfected with Flag-GSDMD-CT along with the Myc-tagged indicated cargo receptors. **b**, **c**

Coimmunoprecipitation and immunoblot analysis of HEK293T cells transfected with Flag-RIG-I or Flag-TBK1, along with the Myc-tagged indicated cargo receptors. **d**, **e** Coimmunoprecipitation and immunoblot analysis of HEK293T cells transfected with Flag-RIG-I or Flag-TBK1, along with the Myc-tagged indicated cargo receptors, with or without HA-GSDMD-CT. **f** RT-PCR analysis of indicated mRNA levels in the HEK293T cells transfected with control siRNA or NDP52 or TOLLIP siRNA for 24 h. Data are represented as mean ± SD. ***p < 0.001, **p < 0.01 (Student's t test). **g**, **h** HEK293T cells were transfected with control siRNA or NDP52 or TOLLIP siRNA for 24 h, then the cells were transfected with EV or HA-GSDMD-CT, together with Flag-RIG-I or Flag-TBK1 for 24 h. Cell lysates were collected for immunoblot analysis. **i** HEK293T cells were transfected with control siRNA or NDP52 or TOLLIP siRNA for 24 h, then the cells were transfected with EV or HA-GSDMD-CT for 24 h. Cell lysates were collected for immunoblot analysis. **j**, **k** HEK293T cells were transfected with indicated plasmids separately for 24 hours. The corresponding proteins were enriched using beads with specific antibodies. Flag-tagged proteins were eluted from Flag beads using Flag peptide and subsequently incubated with Myc-tagged proteins immobilized on Myc beads. Flag proteins were then detected using an anti-Flag antibody. **l**, **m** HEK293T cells were transfected with control siRNA or NDP52 or TOLLIP siRNA for 24 h, then the cells were transfected with EV or HA-GSDMD-CT for 24 h, followed by VSV infection for 12 h. Indicated gene expression was determined by RT–PCR. Data are represented as mean ± SD. ****p < 0.0001, **p < 0.01, *p < 0.05 (Student's t test).

a

RIG-I-CARD

MTTEQRRSLQAFQDYIRKTLDPTYILSYMAPWFREEEVQYIQAEK
NNKGPMEAATLFLKFLLELQEEGWFRGFLDALDHAGYSGLYEAIE
SWDFKKIEKLEEYRLLLKRLQPEFKTRIIPTDIISDLSECLINQECEEI
LQICSTKGMMAGAEKLVECLLRSDKENWPKTLKLALEKERNKFSE
LWIVEKGIKDVETEDLEDKMETSDIQIFYQEDPECQNLSENSCP

Human	15 YIRKTL	45 KNNKGP	96 KIEKLE	112 PEFKTR	151 GAEKLV	161 RSDKEN
Mouse	15 YIKKIL	45 KNNKGP	96 KIEKLE	112 PEFKAT	151 GAEKMA	161 RSDKEN
Pig	15 YVRKTL	45 KNNKGP	96 KIEKLE	112 PEFKTT	151 GAEKMV	161 RSDKEN

Human	166 NWPKTL	169 KTLKLA	174 ALEKER	178 ERNKFS	187 IVEKGI	190 KGIKDV
Mouse	166 NWPKVL	169 KVLQLA	174 ALEKDN	178 DNSKFS	187 IVDKGF	190 KGFKRA
Pig	166 NWPKTL	169 KTLKLA	174 ALEKEE	178 EESRFS	187 MVDKGA	190 KGAEDV

b

TBK1-CC

REPLNTIGLIYEKISLPKVHPRYDLDGDASMAKAITGVVCYACRIAS
TLLLYQELMRKGIRWLIELIKDDYNETVHKKTEVVITLDFCIRNIEKT
VKVYEKLMKINLEAAELGEISDIHTKLLRLSSSQGTIETSLQDIDSRL
SPGGSLADAWAHQEGTHPKDRNVEKLQVLLNCMTEIYYQFKKDK
AERRLAYNEEQIHKFDKQKLYYHATKAMTHFTDECVKKYEAFLNK
SEEWIRKMLHLRKQLLSLTNQCFDIEEEVSKYQEYTNELQETLPQ
KMFTASSGIKHTMTPIYPSSNTLVEMTLGMKKLKEEMEGVVKELA
ENNHILERFGSLTMDGGLRNVDCL

Human	393 IYEKIS	448 ELIKDD	484 KLMKIN	501 IHTKLL	581 QIHKFD	612 FLNKSE
Mouse	393 RYEKIS	448 ELVKDD	484 KLMKVN	501 IHTKLL	581 QIHKFD	612 FKDKSE
Pig	393 IYEKIS	448 ELVKDD	484 KLMKIN	501 IHTKLL	581 QIHKFD	612 FLDKSE

Human	658 LPQKMF	667 SGIKHT
Mouse	658 LPQKML	667 GGVKHA
Pig	658 LPQKMF	667 SGIKHT

Figure S6. Candidate ubiquitination sites of RIG-I and TBK1, related to Figure 5.

Scheme of human RIG-I (**a**) and TBK1 (**b**) protein marked with four candidate ubiquitination sites. Sequence alignments of these sites within RIG-I and TBK1 orthologues from different species are shown below.

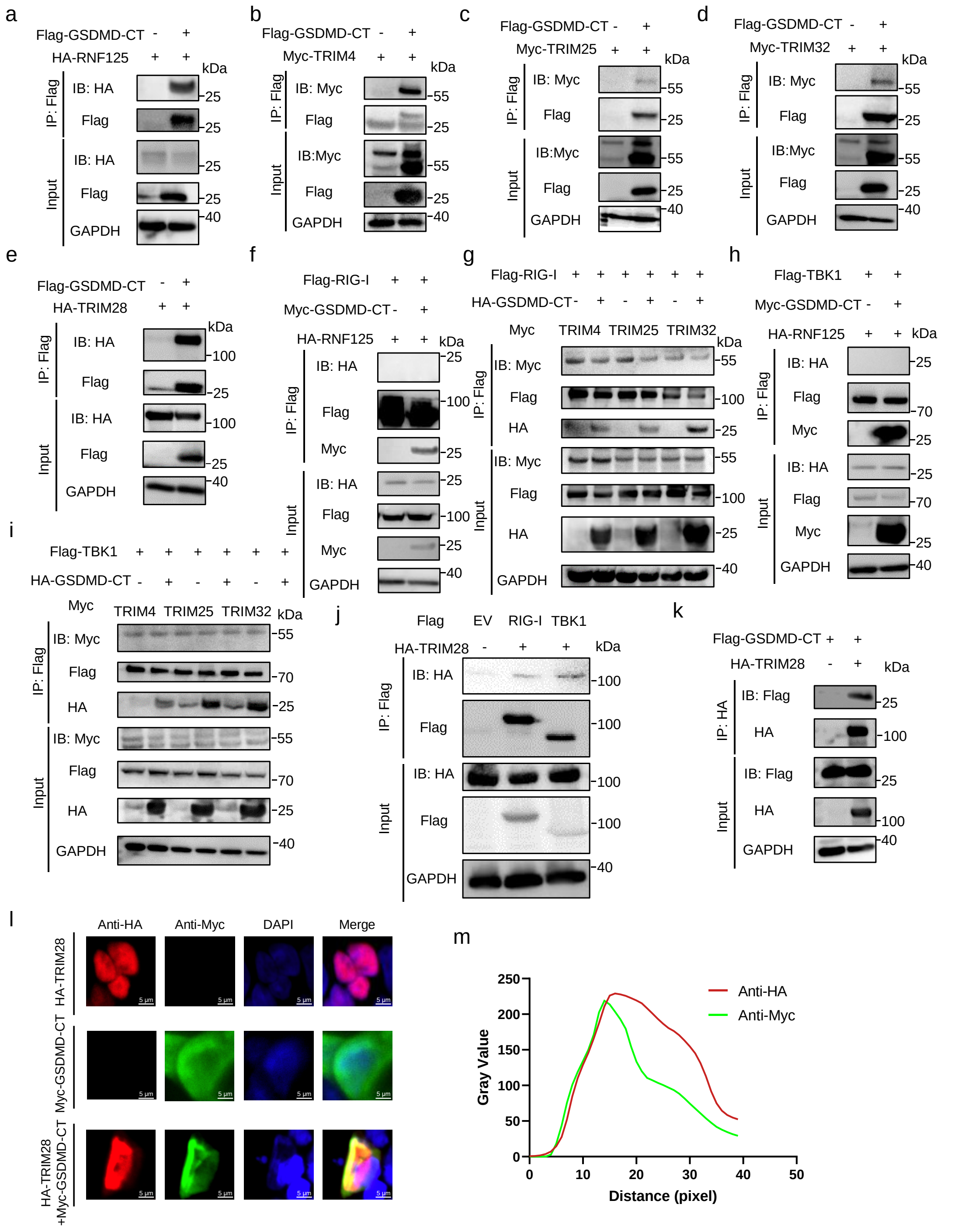

Figure S7. GSDMD-CT faciliates the ubiquitination of RIG-I and TBK1 through the E3 ubiquitin ligase TRIM28, related to Figure 6.

a-e Coimmunoprecipitation and immunoblot analysis of HEK293T cells transfected with Flag-GSDMD-CT and indicated E3 ligases. **f-i** Coimmunoprecipitation and immunoblot analysis of HEK293T cells transfected with indicated E3 ligases and Flag- RIG-I (**f**, **g**) or Flag-TBK1 (**h**, **i**), along with or without Myc-GSDMD-CT. **j**

Coimmunoprecipitation and immunoblot analysis of HEK293T cells transfected with HA-TRIM28 and Flag-RIG-I or Flag-TBK1. **k** Coimmunoprecipitation and immunoblot analysis of HEK293T cells transfected with Flag-GSDMD-CT and HA-TRIM28. **l** Immunofluorescence microscopy and nuclear staining (with the DNA-binding dye DAPI) of HEK293T cells transfected with expression plasmids for HA-TRIM28 and Myc-GSDMD-CT. Scale bars, 5 μm. **m** Colocalization analysis of fluorescent signals of **l**. The Gray Value represents the intensity of the fluorescent signals, while the Distance indicates the spatial separation between the fluorescently labeled structures.

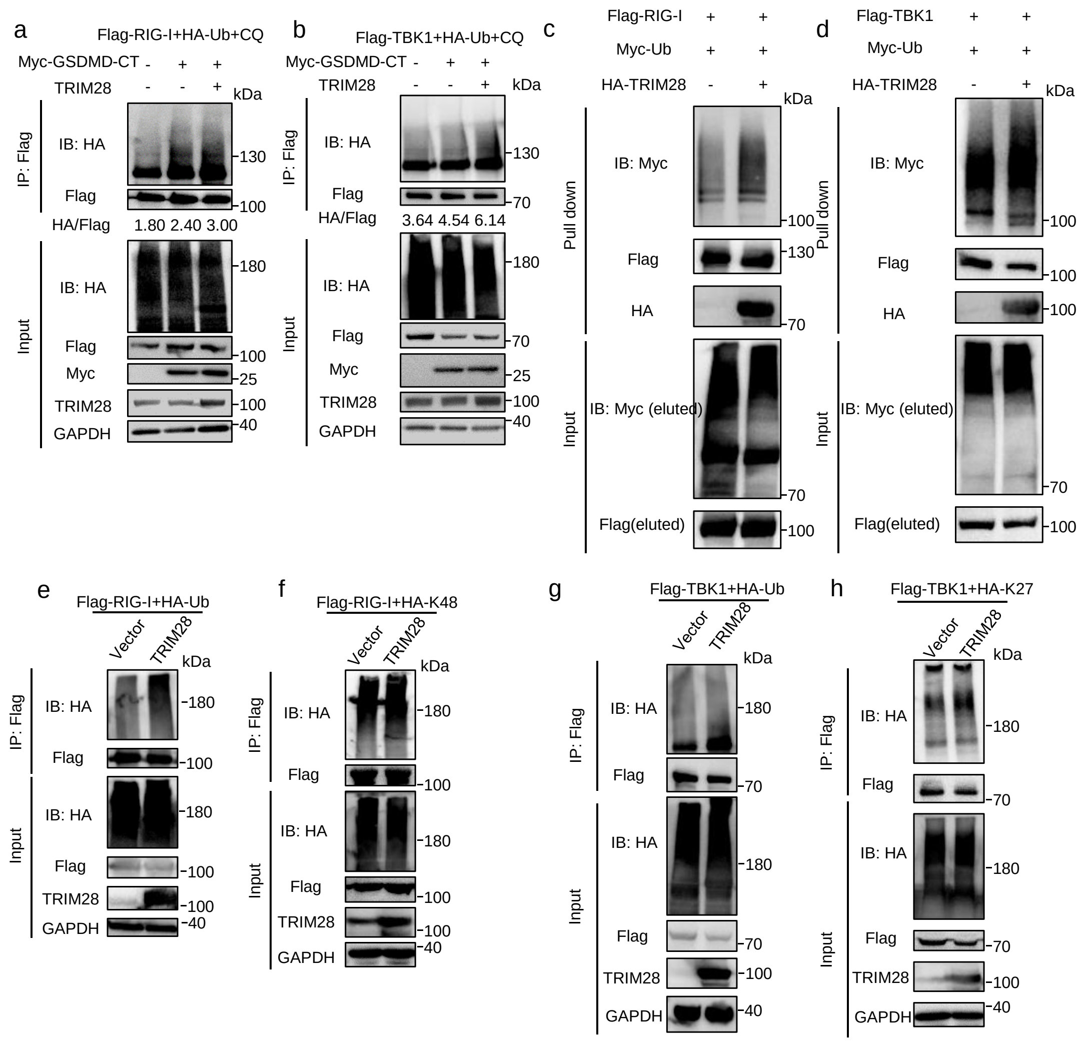

Figure S8. TRIM28 faciliates the ubiquitination of RIG-I and TBK1, related to Figure 6.

a, **b** Coimmunoprecipitation and immunoblot analysis of HEK293T cells transfected with HA-Ub, Myc-GSDMD-CT, HA-TRIM28 and Flag-RIG-I or Flag-TBK1, in the presence of CQ (40 μM) for 6 h. **e-h** Coimmunoprecipitation and immunoblot analysis of HEK293T cells transfected with Flag-RIG-I (**e**, **f**) or Flag-TBK1 (**g**, **h**), along with the TRIM28 and HA-Ub or indicated Ub mutants.

2. Supplementary Tables

Supplementary Table 1. DEGs list

ENSEMBL	log2FoldChange	padj
ENSG00000013588	1.091517	0.004602881
ENSG00000070669	1.208288	8.18E-10
ENSG00000092621	1.588175	3.72E-75
ENSG00000099954	1.002024	0.001291718
ENSG00000100889	1.190385	3.02E-49
ENSG00000101255	1.633337	7.83E-158
ENSG00000101695	1.085874	9.08E-07
ENSG00000101890	1.799598	0.000229613
ENSG00000103257	1.781685	5.60E-173
ENSG00000104518	1.538691	3.57E-57
ENSG00000105281	1.192306	6.48E-66
ENSG00000106105	1.01183	1.75E-44
ENSG00000106113	1.149306	0.041292118
ENSG00000107731	1.338999	4.69E-71
ENSG00000111728	1.86749	0.006523556
ENSG00000111816	1.859877	0.013124302
ENSG00000111981	1.615371	2.45E-13
ENSG00000113739	1.393674	1.68E-65
ENSG00000116761	2.164714	6.29E-60
ENSG00000123685	1.624281	0.000730547
ENSG00000126500	2.082357	5.72E-09
ENSG00000128165	1.757544	4.92E-47
ENSG00000128965	2.055407	3.74E-69
ENSG00000129474	1.647018	7.92E-41
ENSG00000130766	1.620719	1.10E-130

ENSG00000132846	1.132197	0.000166733
ENSG00000135069	1.940602	8.62E-178
ENSG00000135269	1.059341	1.08E-28
ENSG00000136010	1.350143	7.79E-52
ENSG00000139514	1.101142	2.00E-95
ENSG00000140044	1.023285	3.34E-21
ENSG00000141448	1.062657	0.000133506
ENSG00000142405	1.836728	1.28E-16
ENSG00000146733	1.213221	5.78E-39
ENSG00000151150	1.081228	5.00E-06
ENSG00000153714	1.482645	0.000237955
ENSG00000154319	1.297517	2.39E-05
ENSG00000160200	1.574604	1.70E-21
ENSG00000164294	1.030119	2.09E-07
ENSG00000164683	1.350472	1.76E-07
ENSG00000166123	1.331875	3.02E-49
ENSG00000166173	1.412518	0.014807093
ENSG00000166503	1.064632	4.05E-13
ENSG00000167971	1.349535	0.016353607
ENSG00000168297	1.039639	6.20E-43
ENSG00000168672	1.161016	1.89E-13
ENSG00000169136	1.098897	7.21E-34
ENSG00000174136	1.021334	1.14E-19
ENSG00000175906	1.016353	3.37E-08
ENSG00000176046	1.637136	0.019194317
ENSG00000182557	1.561417	0.003945737
ENSG00000182580	1.236977	0.01218584
ENSG00000183010	1.273445	4.07E-20
ENSG00000196159	1.260983	0.009901332

ENSG00000196517	2.856993	1.55E-35
ENSG00000198142	1.019361	0.000317114
ENSG00000198796	1.191451	7.70E-05
ENSG00000205155	1.030868	0.027070949
ENSG00000217130	2.131778	8.93E-07
ENSG00000236404	1.623724	3.05E-06
ENSG00000240376	1.41034	7.88E-06
ENSG00000243978	2.168999	0.000360745
ENSG00000246763	1.134122	3.39E-05
ENSG00000250539	2.071982	0.000921136
ENSG00000259933	1.010095	0.041915596
ENSG00000261824	1.018835	1.26E-27
ENSG00000274026	1.612624	0.005955486
ENSG00000274536	1.491297	0.001209974
ENSG00000274750	1.36039	0.009597411
ENSG00000278771	1.560661	0.026671696
ENSG00000003137	-1.21586	6.10E-06
ENSG00000004468	-1.32175	6.84E-14
ENSG00000006210	-1.17827	0.001784688
ENSG00000007171	-1.35549	3.28E-08
ENSG00000018625	-1.24318	0.001907802
ENSG00000024526	-1.02006	2.88E-05
ENSG00000047936	-1.64543	0.003467888
ENSG00000050730	-1.79049	0.000614808
ENSG00000066279	-1.17841	4.22E-16
ENSG00000073756	-1.69274	2.64E-72
ENSG00000078081	-1.76612	2.72E-06
ENSG00000078098	-1.20236	5.17E-06
ENSG00000078401	-1.5282	1.24E-10

ENSG00000080573	-1.20671	0.00113648
ENSG00000081041	-1.37942	7.45E-82
ENSG00000102755	-1.34266	1.04E-87
ENSG00000102794	-1.69842	0.000162818
ENSG00000102962	-1.80904	0
ENSG00000104368	-1.53289	0.003742509
ENSG00000105711	-1.22243	5.63E-07
ENSG00000105855	-1.01884	2.06E-14
ENSG00000106178	-1.60973	6.78E-13
ENSG00000106952	-1.68963	0.007292468
ENSG00000108342	-4.28192	1.40E-15
ENSG00000108688	-1.65305	0.000248472
ENSG00000108691	-1.48168	8.42E-28
ENSG00000108702	-1.83004	1.31E-165
ENSG00000110436	-1.92344	3.83E-113
ENSG00000110944	-1.15645	4.53E-06
ENSG00000112984	-1.1108	5.56E-10
ENSG00000114455	-1.54684	1.33E-05
ENSG00000115008	-1.15869	9.08E-36
ENSG00000115009	-1.26384	1.96E-131
ENSG00000115155	-1.17637	0.01861591
ENSG00000115590	-1.2571	0.000625637
ENSG00000117650	-1.11807	0.000141772
ENSG00000118193	-1.01801	4.43E-09
ENSG00000121621	-1.01485	0.000708815
ENSG00000121957	-1.08297	1.51E-08
ENSG00000122641	-1.11285	1.76E-73
ENSG00000123358	-1.19476	0.004594016
ENSG00000124875	-1.29199	8.26E-28

ENSG00000125845	-1.12708	1.71E-09
ENSG00000126353	-1.27429	4.07E-21
ENSG00000126787	-1.23199	3.00E-11
ENSG00000128342	-1.12237	2.06E-33
ENSG00000128536	-1.32255	0.044831834
ENSG00000131979	-1.00675	4.08E-22
ENSG00000132639	-1.20248	5.13E-24
ENSG00000135451	-1.06188	0.000259413
ENSG00000136688	-1.51129	0.022012691
ENSG00000137033	-1.77567	4.17E-20
ENSG00000137441	-1.74086	4.85E-06
ENSG00000137507	-1.0296	0.0003064
ENSG00000137868	-1.10102	0.013723819
ENSG00000138771	-1.61143	2.14E-05
ENSG00000144395	-1.00062	0.003262526
ENSG00000147655	-1.37057	0.049767926
ENSG00000148344	-1.68855	5.51E-05
ENSG00000148677	-1.5593	1.68E-101
ENSG00000148848	-1.31533	4.12E-11
ENSG00000149968	-1.85499	2.09E-57
ENSG00000150637	-1.17468	0.001092322
ENSG00000154175	-1.48754	1.18E-05
ENSG00000154864	-1.63161	3.38E-06
ENSG00000157445	-1.21323	0.010812079
ENSG00000159167	-1.20294	2.62E-137
ENSG00000161835	-1.32979	0.017759886
ENSG00000163631	-1.09455	0.031949581
ENSG00000163734	-1.02702	3.08E-41
ENSG00000163735	-1.13749	5.11E-09

ENSG00000163739	-2.04304	3.76E-184
ENSG00000163874	-1.33291	4.36E-54
ENSG00000164070	-1.17229	8.98E-16
ENSG00000164171	-1.40074	0.00028835
ENSG00000165474	-1.9007	2.10E-09
ENSG00000165507	-1.60187	8.03E-29
ENSG00000166396	-1.33772	0.000806907
ENSG00000167034	-1.08144	2.44E-10
ENSG00000168398	-1.85513	6.18E-12
ENSG00000169429	-1.02581	1.12E-41
ENSG00000170054	-3.23492	9.47E-37
ENSG00000171303	-1.08564	0.008915613
ENSG00000171502	-1.42092	0.004393343
ENSG00000172179	-1.84022	0.000618356
ENSG00000173227	-1.39486	5.33E-05
ENSG00000179954	-1.25731	0.04198136
ENSG00000180616	-1.24936	0.000613943
ENSG00000181374	-2.2738	1.06E-06
ENSG00000182162	-1.11612	2.93E-10
ENSG00000187627	-1.37096	0.000667982
ENSG00000197632	-1.80769	1.63E-05
ENSG00000198771	-1.08561	1.80E-09
ENSG00000204389	-1.07195	0.000688659
ENSG00000204580	-1.365	1.06E-08
ENSG00000205755	-1.53123	4.03E-15
ENSG00000206073	-4.51647	1.66E-06
ENSG00000228705	-1.19037	0.002603744
ENSG00000232810	-1.08994	8.10E-63
ENSG00000233237	-1.3508	0.013252611

ENSG00000234511	-1.00414	0.00736495
ENSG00000236714	-2.04893	0.001606676
ENSG00000240280	-1.56072	0.012700718
ENSG00000253522	-1.1239	9.78E-05
ENSG00000255823	-1.28239	0.003193137
ENSG00000263513	-1.05969	0.027298882
ENSG00000274276	-2.01094	0.010364673
ENSG00000275302	-1.98422	1.29E-124
ENSG00000276070	-2.12765	2.65E-212
ENSG00000276070	-2.12765	2.65E-212
ENSG00000276085	-1.2602	1.95E-97
ENSG00000276085	-1.2602	1.95E-97
ENSG00000277632	-1.40432	1.11E-112
ENSG00000285077	-1.2982	0.017895539

Supplementary Table 2. Residues and bond lengths involved in hydrogen bond formation for GSDMD-CT and RIG-I.

residue and atom of RIG-I		residue and atom of GSDMD-CT		Distances of Hydrogen bond
208SER	OG	295GLU	O	4
209SER	O	295GLU	OE1	3.7
211GLN	OE1	290GLN	N	4.1
211GLN	NE2	288ASP	N	3.7
211GLN	OE1	291GLY	N	3.5
211GLN	N	292LEU	N	3
211GLN	O	470SER	OG	4.1
212ILE	O	287ALA	N	3.7
213PHE	O	419GLN	NE2	3.4
501GLN	OE1	290GLN	NE2	4
512GLN	OE1	286ALA	N	3.1
899VAL	O	337GLN	N	4.1

Supplementary Table 3. Residues and distances involved in hydrophobic interactions for GSDMD-CT and RIG-I.

residue and atom of RIG-I		residue and atom of GSDMD-CT		Distances of Hydrophobic
210ILE	CG2	292LEU	CD1	2.4
210ILE	CD1	296VAL	CG2	3.1
210ILE	CG1	295GLU	CB	1.8
212ILE	CG2	466LEU	CG	2.3
212ILE	CB	287ALA	CB	4.0
213PHE	CE2	288ASP	CB	3.9
213PHE	CD1	419GLN	CG	2.6
213PHE	CD2	418TRP	CE3	2.4
213PHE	CB	466LEU	CD2	3
214ILE	CD1	467ILE	CD1	2.5
215GLN	CB	463PRO	CB	3.7
512GLN	CB	286ALA	CB	3.2
513TRP	CB	286ALA	CB	3.8
899VAL	CG1	333ALA	CB	2.7
902VAL	CG2	375PHE	CZ	2.3

Supplementary Table 4. Residues and bond lengths involved in hydrogen bond formation for GSDMD-CT and TBK1.

residue and atom of TBK1		residue and atom of GSDMD-CT		Distances of Hydrophobic
401LYS	NZ	419GLN	NE2	3.7
577TYR	OH	302GLU	N	4
578ASN	ND2	369GLU	OE1	3.9
584LYS	NZ	295GLU	O	3.9
588GLN	NE2	376TYR	OH	3.6

Supplementary Table 5. Hydrophobic interactions formed between GSDMD-CT and TBK1.

residue and atom of TBK1		residue and atom of GSDMD-CT		Distances of hydrophobic interactions
402ILE	CB	467ILE	CG1	3.8
402ILE	CG1	467ILE	CD1	3.5
403HIS	CB	287ALA	CB	3.9
577TYR	CE1	302GLU	CG	3.9
578ASN	CB	368PRO	CB	3.4
581GLN	CB	372ALA	CB	2.6
582ILE	CG1	339LEU	CD1	3.4
582ILE	CD1	339LEU	CB	3.0
584LYS	CD	295GLU	CG	3.8
584LYS	CB	376TYR	CD2	2.9
585PHE	CG	376TYR	CA	2.2
585PHE	CD2	375PHE	CB	2.9
585PHE	CB	375PHE	CD2	2.4
589LYS	CE	375PHE	CZ	3.5
591TYR	CE1	467ILE	CG2	2
591TYR	CE1	467ILE	CD1	2.9
591TYR	CD1	383VAL	CG2	3.8

Supplementary Table 6. Primers used in this study for qPCR in human

Name	Sense (5'-3')
RIG-I-F	TGTGCTCCTACAGGTTGTGGA
RIG-I-R	CACTGGGATCTGATTCGCAAAA
TBK1-F	TCGCTGACTAATCAGTGTTTTGA
TBK1-R	GGGTCATGGTATGTTTGATTCC
GAPDH-F	AGCCACATCGCTCAGACAC
GAPDH-R	GCCCAATACGACCAAATCC
Isg54-F	TATTGGTGGCAGAAGAGGAAGA
Isg54-R	CAGGTGAAATGGCATTTTAGTT
Isg56-F	TCAGGTCAAGGATAGTCTGGAG
Isg56-R	AGGTTGTGTATTCCCACACTGTA
NDP52-F	AGATGAAGGAGGCGCAAGAC
NDP52-R	TCTGCTTTGCAGATAGGGCA
TOLLIP-F	ACAAGTGGTACAGCCTGAGC
TOLLIP-R	TGCTGGTACACTGTTGGCAT
TRIM28-F	CTCGGGATGGTGAACGTACT
TRIM28-R	GCAATGTTGCATGTTTGTCC
IFNB-F	CTTTGCTATTTTCAGACAAGATTCA
IFNB-R	GCCAGGAGGTTCTCAACAAT

Supplementary Table 7. Primers used in this study for qPCR in mouse

Name	Sense (5’-3’)
IFNB-F	TCCTGCTGTGCTTCTCCACCACA
IFNB-R	AAGTCCGCCCTGTAGGTGAGGTT
Isg15-F	GGTGTCCGTGACTAACTCCAT
Isg15-R	CTGTACCACTAGCATCACTGTG
Isg56-F	TGCGATCCACAGTGAACAAC
Isg56-R	ACTTCCGGGAAATCGATGAG
GAPDH-F	AAGGTCATCCCAGAGCTGAA
GAPDH-R	CTGCTTCACCACCTTCTTGA

Supplementary Table 8. Primers used in this study for virus detection

Name	Sense (5’-3’)
VSV-F	TGGGATGACTGGGCTCCATA
VSV-R	CACCATCAGGAAGCTGCGAA
EMCV-F	TGAGTCATTAGCCATTTCAACCCA
EMCV-R	CGTGAGATACAAACCCGCCCTA

Supplementary Table 9. siRNA sequences

Name	Sense (5’-3’)
si-NDP52-F	GGACGUCACAUGUCAUUAUTT
si-NDP52-R	AUAAUGACAUGUGACGUCCTT
si-Tollip-F	GCUGGAAUAAGGUCAUCCATT
si-Tollip-R	UGGAUGACCUUAUUCCAGCTT
si-TRIM28-F	GCAUGAACCCCUUGUGCUGTT
si-TRIM28-R	CAGCACAAGGGGUUCAUGCTT

Supplementary Table 10. Key resources table.

REAGENT or RESOURCE	SOURCE	IDENTIFIER
Antibodies		
Rabbit anti GSDMD	Cell Signaling	Cat#39754; RRID: AB_2916333
Rabbit anti GSDMD-NT	Abcam	Cat#ab215203; RRID: AB_2916166
Rabbit anti GSDMD-CT	Abcam	Cat#ab227821
Rabbit anti GSDMD-CT	ABclonal	Cat#A23755
Mouse anti GAPDH	ABclonal	Cat#AC002, RRID: AB_2736879
Rabbit anti pTBK1	Abcam	Cat#ab109272, RRID: AB_10862438
Rabbit anti TBK1	Cell Signaling	Cat#3504, RRID: AB_2255663
Mouse anti Flag	Sigma-Aldrich	Cat#F1804; RRID: AB_262044
Mouse anti Flag	ABclonal	Cat#AE005, RRID: AB_2770401
Rabbit anti pIRF3	Abcam	Cat#ab76493, RRID: AB_1523836
Rabbit anti IRF3	Cell Signaling	Cat#11904; RRID: AB_2722521
Rabbit anti HA	Cell Signaling	Cat#3724, RRID: AB_1549585
Rabbit anti Myc	Sigma-Aldrich	Cat#C3956, RRID: AB_439680
Rabbit anti ATG5	Huabio	Cat#ET1611-38, RRID: AB_3070016
Rabbit anti BECN1	Huabio	Cat#HA721216, RRID: AB_3072336
Rabbit anti RIG-I	ABclonal	Cat#A0550, RRID: AB_2757259
Rabbit anti NDP52	Huabio	Cat#HA721356, RRID: AB_3072473
Rabbit anti TOLLIP	Huabio	Cat#ER65194
Rabbit anti TRIM28	Huabio	Cat#ET1612-55, RRID: AB_3070129
Goat anti-Mouse Alexa Fluor 488	Abcam	Cat#ab150113, RRID: AB_2576208
Goat anti-Mouse Alexa Fluor 555	Abcam	Cat#ab150114, RRID: AB_2687594
Donkey anti-Rabbit Alexa Fluor 647	Abcam	Cat#ab150075, RRID: AB_2752244
Goat Anti-Rabbit IgG(H+L)-HRP	Fude Biotech	Cat#FDR007, RRID: AB_2934270
Goat Anti-Mouse IgG(H+L)-HRP	Fude Biotech	Cat#FDM007, RRID: AB_2934269
Bacterial and virus strains		
VSV	Shu Zhu Lab	N/A

HSV	Shu Zhu Lab	N/A
EMCV	Shu Zhu Lab	N/A
SeV	Jiyong Zhou Lab	N/A
Chemicals, peptides, and recombinant proteins		
PMA	Sigma-Aldrich	Cat#P1585
Nigericin	InvivoGen	Cat#tlrl-nig
Poly(dA:dT)	InvivoGen	Cat#TLRL-PATN
Poly(I:C)	Millipore	Cat#528906
MG132	APExBIO	Cat#A2585
3-Methyladenine (3MA)	MCE	Cat#HY-19312
Chloroquine (CQ)	Sigma	Cat# C6628
Cycloheximide (CHX)	MCE	Cat#HY-12320
murine M-CSF	Peprotech	Cat#315-02
PVDF membranes	Bio-rad	Cat#1620177
Fetal Bovine Serum	gibco	Cat#10091148
DMEM, high glucose	gibco	Cat#11965500
RPMI1640	gibco	Cat#11875500
FreeZol Reagent	Vazyme	Cat#R711
SYBR qPCR Master Mix	Vazyme	Cat#Q712
HiScript III RT SuperMix for qPCR	Vazyme	Cat#R323
Anti-Flag M2 Magnetic Beads	Sigma-Aldrich	Cat#10004D
Entranster-in vivo	Engreen	Cat#18668-11-2
Critical commercial assays		
Dual-Luciferase Reporter Assay Kit	Beyotime	Cat#RG029
Human IFN-β ELISA kit	MultiSciences	Cat#EK1236
Mouse IFN-β ELISA kit	MultiSciences	Cat#EK2236
CytoTox LDH-release assay kit	Promega	Cat#G1780
Experimental models: Cell lines		
Human: HEK293T cells	Shi et al.,2022	N/A

Human: THP-1 cells	Shi et al.,2022	N/A
Human: THP-1 GSDMD KO cells	Shi et al.,2022	N/A
Human: ATG5-KO 293T cells	Jun Cui Lab	N/A
Human: BECN1-KO 293T cells	Jun Cui Lab	N/A
Experimental models: Organisms/strains		
Mouse: GSDMD-KO C57BL/6J	GemPharmatech	N/A
Oligonucleotides		
Table S6	This paper	N/A
Table S7	This paper	N/A
Table S8	This paper	N/A
Table S9	This paper	N/A
Recombinant DNA		
TK	Boli Hu Lab	N/A
IFN-β luciferase	Boli Hu Lab	N/A
Flag-GSDMD-FL	Shi et al.,2022	N/A
Flag-GSDMD-CT	This paper	N/A
HA-GSDMD-CT	This paper	N/A
Myc-GSDMD-CT	This paper	N/A
Flag-cGAS	This paper	N/A
Flag-STING	This paper	N/A
Flag-MDA5	This paper	N/A
Flag-RIG-I-N	Boli Hu Lab	N/A
Flag-RIG-I	This paper	N/A
Flag-MAVS	This paper	N/A
Flag-TBK1	This paper	N/A
Flag-IRF3	This paper	N/A
Myc-TBK1	This paper	N/A
Myc-RIG-I	This paper	N/A
Myc-TBK1-mutants	This paper	N/A

Myc-RIG-I-mutants	This paper	N/A
Flag-TBK1-mutants	This paper	N/A
Flag-RIG-I-mutants	This paper	N/A
Myc-SQSTM1	This paper	N/A
Myc-NDP52	This paper	N/A
Myc-NBR1	This paper	N/A
Myc-TAX	This paper	N/A
Myc-OPTN	This paper	N/A
Myc-TOLLIP	This paper	N/A
Myc-NIX	This paper	N/A
HA-Ub	Shi et al.,2022	N/A
HA-Ub-mutants	Shi et al.,2022	N/A
HA-TRIM28	This paper	N/A
HA-RNF125	This paper	N/A
Myc-TRIM4	This paper	N/A
Myc-TRIM25	This paper	N/A
Myc-TRIM32	This paper	N/A
Flag-GSDMs-CT	This paper	N/A
Software and algorithms		
GraphPad Prism	GraphPad Software	https://www.graphpad.com/
Image J	NIH	https://imagej.nih.gov/ij/
